# Diversity of *Leptogium* (Collemataceae, Ascomycota) in East African Montane Ecosystems

**DOI:** 10.3390/microorganisms9020314

**Published:** 2021-02-03

**Authors:** Ulla Kaasalainen, Veera Tuovinen, Paul M. Kirika, Neduvoto P. Mollel, Andreas Hemp, Jouko Rikkinen

**Affiliations:** 1 Department of Geobiology, University of Göttingen, Goldschmidtstraβe 3, 37077 Göttingen, Germany; 2 Finnish Museum of Natural History, P.O. Box 7, University of Helsinki, 00014 Helsinki, Finland; jouko.rikkinen@helsinki.fi; 3 Department of Ecology and Genetics, Uppsala University, Norbyvägen 18D, 752 36 Uppsala, Sweden; veera.tuovinen@ebc.uu.se; 4 National Museums of Kenya, East African Herbarium, Museum Hill Road, P.O. Box 45166, Nairobi 00100, Kenya; pkirika@museums.or.ke; 5 National Herbarium, Tropical Pesticides Research Institute, P.O. Box 3024, Arusha 23201, Tanzania; neduvoto.mollel@tpri.go.tz; 6 Department of Plant Systematics, University of Bayreuth, Universitätsstr. 30, 95440 Bayreuth, Germany; andreas.hemp@uni-bayreuth.de; 7 Organismal and Evolutionary Biology Research Programme, Faculty of Biological and Environmental Sciences, University of Helsinki, P.O. Box 65, 00014 Helsinki, Finland

**Keywords:** biodiversity hotspot, Mount Kilimanjaro, Taita Hills, Mount Kasigau

## Abstract

Tropical mountains and especially their forests are hot spots of biodiversity threatened by human population pressure and climate change. The diversity of lichens in tropical Africa is especially poorly known. Here we use the mtSSU and nuITS molecular markers together with morphology and ecology to assess *Leptogium* (Peltigerales, Ascomycota) diversity in the tropical mountains of Taita Hills and Mt. Kasigau in Kenya and Mt. Kilimanjaro in Tanzania. The sampled habitats cover a wide range of ecosystems from savanna to alpine heath vegetation and from relatively natural forests to agricultural environments and plantation forests. We demonstrate that *Leptogium* diversity in Africa is much higher than previously known and provide preliminary data on over 70 putative species, including nine established species previously known from the area and over 60 phylogenetically, morphologically, and/or ecologically defined Operational Taxonomic Units (OTUs). Many traditional species concepts are shown to represent morphotypes comprised of several taxa. Many of the species were only found from specific ecosystems and/or restricted habitats and are thus threatened by ongoing habitat fragmentation and degradation of the natural environment. Our results emphasize the importance of molecular markers in species inventories of highly diverse organism groups and geographical areas.

## 1. Introduction

Tropical mountains and especially the montane forests are hot spots of biodiversity and endemism and may represent true evolutionary cradles especially for neoendemics [1,2,3,4,5]. The tropical rainforests of eastern Africa originated approximately 30 million years ago and have persisted through climatic fluctuations, mainly due to the atmospheric moisture supplied by remarkably stable Indian Ocean currents [6]. There, a very complex climatic history has fragmented a once extensive ancient forest ecosystem and given rise to many unique habitats with high levels of local endemism [1,2,5,6,7]. The windward slopes of many East African mountains benefit from moisture brought by the trade winds and sustain the last remaining fragments of East African montane rain forests, surrounded by more extensive arid forests and woodlands [6].

The Taita Hills in south-eastern Kenya form the northernmost section of the ancient Eastern Arc Mountains. The evergreen montane forests on the Eastern Arc are probably the oldest remaining forests in East Africa, and they effectively link the forests of the Indian Ocean coast to the tropical forests of central Africa and the younger volcanic mountains of the Rift Valley [8,9]. Together with the coastal forests of Tanzania and Kenya, the montane forests of the Taita Hills represent a hotspot of global biodiversity with a high number of endemic vertebrates and plants [1,10,11,12]. On the other hand, the much younger volcanic mountains, including Mt. Kilimanjaro, also support high concentrations of biodiversity due to the steep environmental gradients of their slopes. Even though the level of endemism on Mt. Kilimanjaro is believed to be lower than on that of nearby Eastern Arc Mountains, this may more reflect the results of anthropogenic influence and the destruction of lower-montane forests, rather than the relatively young age of the mountain [13].

Tropical forests and their biodiversity worldwide are threatened by human population pressure and climate change [14,15]. Especially in sub-Saharan Africa, the forest loss proceeds at an alarming rate and is particularly severe in the Afromontane areas [16]. For example, in the Taita Hills the indigenous forest area decreased 50% just between 1955 and 2004, and the remaining forests have suffered substantial degradation due to agricultural expansion [17]. The forests of East Africa are already among the most threatened regions of global biodiversity and extinction risk for many organisms is still increasing [18,19]. On Mt. Kilimanjaro, forest corridors to nearby mountains have vanished due to deforestation, leaving the mountain isolated [5]. At the higher altitudes, the montane ecosystems are influenced for example by climate change-driven forest fires [20] and altogether, Kilimanjaro has lost about 50% of its forest cover since the beginning of the last century [13]. In general, habitat destruction is the leading cause of species extinction and this is especially true in the tropics [21,22]. For example, tropical epiphytes are severely threatened by deforestation and disturbance [23,24]. It is suspected that numerous plant and animal species, including many of the local endemics, currently experience great difficulties in maintaining stable populations in the highly fragmented forest landscape of the Taita Hills [25,26,27]. This is probably true also for epiphytic lichens and bryophytes, but so far, no studies have dealt with this issue. Even on a general level, the lichen flora of tropical Africa is still very poorly known.

*Leptogium* (Ach.) Gray (Collemataceae, Ascomycota) is a genus of approximately a hundred foliose, mainly epiphytic macrolichen species with a nearly cosmopolitan but predominately humid temperate and tropical distribution [28]. The application of molecular phylogenetic methods has led to the revision of Collemataceae and re-evaluation of the taxonomic value of many morphological characters [28,29,30]. For example, a number of species previously placed in *Leptogium* were moved into their own genus, *Scytinium* (Ach.) Gray, while some taxa from other genera have transferred into *Leptogium* [28,31]. In 1988, Swinscow and Krog [32] listed 24 *Leptogium* species from East Africa, including collections from Ethiopia, Kenya, Tanzania, and Uganda. Since then, few further species inventories dealing with *Leptogium* species have been made in the region [33,34,35]. However, one such study recently demonstrated that East African specimens previously identified as *Leptogium hibernicum* M. E. Mitch. ex P. M. Jørg. actually represent a separate lineage, *L. krogiae* Bjelland, Frisch & Bendiksby, so far only known from East Africa [36]. In addition, *Leptogium ethiopicum* C.W. Dodge (1964), for a time considered a synonym of *L. burgessii*, was reinstated as a separate species [37]. In conclusion, 25 *Leptogium* species are currently listed from East Africa, these including: *L. adpressum* Nyl., *L. asiaticum* P.M. Jørg., *L. austroamericanum* (Malme) C.W. Dodge, *L. azureum* (Sw.) Mont., *L. brebissonii* Mont., *L. burgessii* (L.) Mont., *L. burnetiae* C.W. Dodge, *L. caespitosum* (Taylor) Swinscow & Krog, *L. cochleatum* (Dicks.) P.M. Jørg. & P. James, *L. coralloideum* (Meyen & Flot.) Vain., *L. cyanescens* (Ach.) Körb., *L. digitatum* (A. Massal.) Zahlbr., *L. ethiopicum*, *L. furfuraceum* (Harm.) Sierk, *L. javanicum* Mont., *L. juressianum* Tav., *L. krogiae, L. laceroides* de Lesd., *L. marginellum* (Sw.) Gray, *L. phyllocarpum* (Pers.) Mont., *L. punctulatum* Nyl., *L. resupinans* Nyl., *L. rivulare* (Ach.) Mont., *L. sessile* Vain., and *L. vesiculosum* (Sw.) Malme [32,33,36]. Many of these taxa are currently perceived to have wide distributions in the tropics and in temperate regions, and the types of a vast majority have been described from localities outside Africa.

Here, we examine the diversity and habitat ecology of *Leptogium* on several East African mountains, including the Taita Hills and Mt. Kasigau in Kenya and Mt. Kilimanjaro in Tanzania. The study is mostly based on new specimens collected by the authors in 2009–2017 from East Africa.

## 2. Materials and Methods

### 2.1. Study Area and Sampling

The Taita Hills and the neighboring Mt. Kasigau in SW Kenya and Mt. Kilimanjaro in NW Tanzania are three isolated mountain blocks all situated less than 400 km south of the Equator. They are separated by semiarid plains at approximately 600 m above sea level, where the climate is tropical with two distinct rainy seasons. While the amount of precipitation and temperature vary with altitude, the windward southern slopes of the mountains benefit from moisture brought by the trade winds from the Indian Ocean, supporting evergreen montane “cloud forest”. The moist and relatively cool local climate of the montane forest ecosystems provides favorable conditions for the development of diverse lichen and bryophyte communities and abundant epiphyte biomass.

The Taita Hills region includes three closely situated massifs (Dabida, Mbololo, Sagalla) and Mt. Kasigau which is located further away. The mountains rise abruptly from the surrounding plains to a series of ridges, reaching 2208 m at the highest peak Vuria in Dabida. The annual precipitation usually varies between 600 and 1500 mm, but the rainfall on the lowlands at the base of Mt. Kasigau averages only 300–500 mm. On the other hand, some parts of the upper slopes may receive over 2000 mm of atmospheric moisture annually as a combination of rain and mist [9,25,38]. The potential natural vegetation on the upper slopes of the Dabida massif consists of evergreen moist montane forest classified as *Ocotea* forest, with *Cola-Craibia* forest in the drier parts of Ngangao and Mbololo and *Erica-Maesa* forest near the summit of Vuria [39,40]. However, long-lasting and intensive human influence has split the indigenous forest into small, isolated patches and none are in pristine condition: most are heavily disturbed, surrounded, or mixed with exotic plantation trees (e.g., *Acacia mearnsii, Cupressus lusitanica, Grewillea robusta,* and *Pinus* and *Eucalyptus* species) and embedded in an intensively used agricultural landscape [10,17,39]. For example, all mature individuals of the keystone tree species *Ocotea usambarensis* and *Podocarpus latifolius* have been extracted for timber and are presently only seen in the lower canopy, while the forests are dominated by tree species more typical for early succession and with little commercial value or practical use (e.g., *Tabernaemontana stapfiana* and *Phoenix reclinata*) [10,25,40,41]. In general, the smallest fragments of indigenous forest are most affected by disturbance and presently harbor relatively few woody species [25].

The largest remnants of indigenous closed canopy forest on the Dabida massif include: Ngangao (03°21′ S, 38°20′ E, 1750–1900 m alt.), a relatively large (120 ha) and partly less disturbed forest with abundant indigenous species including for example *Albizia gummifera*, *Tabernaemontana stapfiana*, *Newtonia buchananii*, *Strombosia scheffleri*, and *Macaranga capensis*, and *Cola greenwayi* and *Craibia zimmermannii* in the drier parts of the forest [40,41,42]; Chawia (3°28′ S, 38°20′ E, 1500–1600 m alt.), a heavily disturbed forest area of 111 ha including plantations of exotic trees and 86 ha of indigenous forest with for example *Tabernaemontana stapfiana*, *Albizia gummifera*, *Syzygium sclerophyllum*, *Strombosia scheffleri*, and *Phoenix reclinata* [17,41,42]; Vuria (3°25′ S, 38°17′ E, 2000–2200 m alt.), with approximately 100 ha of heavily disturbed forest with less than 1 ha of indigenous forest which however partially represents very dense and humid cloud forest [42,43]; Fururu (3°25′ S, 38°20′ E, 1650–1750 m alt.), Macha (03°25′ S, 38°21′ E, 1600 m alt.), Mwachora (03°25′ S, 38°22′ E, 1650 m alt.), and Yale (03°24′ S, 38°20′ E, 1850 m alt.), the first with 8 ha and the rest approximately 2 ha of indigenous forest each, are all heavily disturbed and intermixed with exotic plantations and agricultural land with indigenous species such as *Tabernaemontana stapfiana*, *Phoenix reclinata*, and *Maesa lanceolata* [41,42]. In addition to these protected forests, there are numerous minute patches of indigenous forest vegetation, like the one on Shomoto Hill with less than 0.2 ha of forest and just a few individual indigenous trees.

Mt. Kasigau (3°30′ S, 38°39′ E), some 50 km SE from the Taita Hills, rises very steeply from the surrounding plains in 600 m to the summit at 1641 m. Starting as an *Acacia-Commiphora* bushland of the surrounding plains, the vegetation transitions through lower montane and *Euphorbia* woodlands and riverine forests (600–1000 m alt.), to semievergreen woodland (890–1250 m), evergreen forest (1086–1380 m), and finally to cloud forest (> 1470 m) [44,45]. The woody flora of Mt. Kasigau represents a special mix of Somalia-Masai, Afromontane and Coastal floristic affinities [45]. Additionally, the Mt. Kasigau forest has remained relatively undisturbed and also conserved a high coverage of the lower-elevation woodlands [45], offering a relatively unique and invaluable continuous transition of vegetation from the surrounding savanna to montane forest. Thus, the continuous forest and woodland area of Mt. Kasigau is significantly larger than the protected evergreen montane forest (203 ha). 

Mt. Kilimanjaro is a relatively young dormant volcano of less than one million years old. The highest peak is almost six kilometers high and 4877 m higher than the surrounding savanna; the remaining forest zones of the mountain are now mainly protected as a part of the Kilimanjaro National Park. The height provides for a huge range of natural vegetation types; however, the pressure of human population has led to increasing water demands, illegal logging, and grazing pressure [46]. For example, as in Taita Hills, *Ocotea usambarensis* is extinct from many parts of the natural *Ocotea* forest, and the upper forest boundary has lowered significantly due to fire [13,20]. In the lower elevations, natural savanna, dry woodlands, and lower montane forest have for a large part been converted into agriculture [5,47]. As a result, the slope of Mt. Kilimanjaro supports several prominent natural and human-modified ecosystems, of which the following were sampled for this study: natural savanna and maize fields (800–1100 m); lower montane forests, traditional Chagga home gardens, commercial coffee farms, and grasslands (1100–2000 m); montane *Ocotea* forest and selectively logged *Ocotea* forest (2100–2800 m); upper montane *Podocarpus* forest and *Podocarpus* forest replaced by *Erica excelsa* forest as a result of fire (2800–3100 m); subalpine *Erica trimera* forest and fire disturbed *Erica* forest (3500–4000 m); and alpine *Helichrysum* heaths (4000–4600 m). Between the ecosystems, the mean annual temperature varies from 23 °C at the base of the mountain to 4 °C at the alpine zone, and night frosts occur above 2700 m [48,49]. The relative humidity and precipitation are highest in the montane forest zones within the stable cloud condensation belt (mean annual precipitation over 2000–2400 mm, partly up to 3000 mm) from where the precipitation diminishes both up to the *Helichrysum* heath (~1300 mm) and down to the savanna (~700 mm) [48]. 

The sampling of lichens in the Taita Hills has taken place during several field trips mainly in 2009–2010, covering most of the described forest fragments on the Dabida massif and the nearby areas; in addition to the forest fragments, also the small garden of the University of Helsinki’s Taita Research Station in approximately 1200 m alt. and nearby roadside trees have been sampled. On Mt. Kasigau, most of the specimens were collected during fieldwork in 2010, when four transects were sampled along the northern, southern, eastern, and western slopes of the mountain. The transects reached from the dry woodland on the base of the mountain to the summit with collection plots in approximately 50 m intervals; a map of the research setting is presented by Enrooth et al. [50]. The sampling in the Kilimanjaro area was done in 2016–2017 along five replicate transects on the southern and southeastern slopes of the mountain; the 65 sampling plots represented the above mentioned 13 natural and disturbed ecosystems, with five replicate plots of each ecosystem, as presented by Rutten et al. [47].

### 2.2. Morphological Inspection and Molecular Methods

The *Leptogium* specimens were identified based on morphology and existing literature from the area [32,36]. A small fragment of each lichen specimen was used to extract DNA using the DNeasy Plant Mini Kit (Qiagen AB, Sollentuna, Sweden) or GeneJET Genomic DNA Purificatiom Kit (Fermentas, Helsinki, Finland or Fisher Scientific GmBH, Schwerte, Germany) following the manufacturer’s instructions. After the extraction, fungal nuclear internal transcribed spacer (nuITS: ITS1-5.8S-ITS2) and mitochondrial small subunit 12S (mtSSU) gene sequences were obtained for the phylogenetic analyses. PCR amplification of the fungal ITS gene region was performed with the primers ITS5 and ITS4 [51] using the Dynazyme II DNA polymerase (Finnzymes, Helsinki, Finland) following the protocols in Fedrowitz et al. [52] or by using GoTaq DNA polymerase (Promega GmBH, Walldorf, Germany) as follows: 0.2 mM dNTPs, 0.2 µM of each primer, 0.5 mg/mL BSA, and 0.025 U/µl polymerase in a total volume of 50 µl; with 2 min in 95 °C for initial denaturation, followed by 35 cycles of 45 s in 95 °C, 45 s in 56 °C, and 1 min in 72 °C, and with a final elongation of 5 min in 72 °C. Amplification of the mtSSU region was performed similarly but using the primers mtSSU1 and mtSSU3R [53] and with an annealing temperature of 59 °C. The PCR products were purified using GeneJET PCR purification kit (Fermentas, Helsinki, Finland) or the service was provided by the sequencing company. Sequencing was done using the PCR primers by Macrogen Inc. (Seoul, Korea) and LGC Genomics (Berlin, Germany). After sequencing, the chromatograms of all DNA sequences were checked, edited, and aligned using BioEdit 7.0.9 [54], PhyDE-1 v0.997 [55], and/or CodonCode Aligner (CodonCode Corporation, Centerville, MA, USA). The nuITS and mtSSU sequences obtained from the *Leptogium* specimens are deposited in the NCBI GenBank database. The specimen information, collection localities, and the GenBank accession numbers are listed in Table S1.

### 2.3. Data Analyses

For the first DNA alignment, all newly obtained unique mtSSU variants and the corresponding 5.8S sequence were included; additional sequences were downloaded from the NCBI GenBank database when available. The rare insertions within the mtSSU region as well as the extremely variable ITS1 and ITS2 regions of the nuITS were excluded before the analysis. *Degelia*, *Leciophysma*, *Staurolemma*, *Rostania*, *Scytinium*, and *Collema* were chosen as outgroups, based on existing knowledge about Peltigerales and Collemataceae [29]. The initial alignment was constructed on the MAFFT online service [56] after which it was manually adjusted. The phylogenetic analysis was performed using Bayesian inference and the data partitioned according to the marker region. Substitution models for the analysis were selected using jModelTest2 [57], and GTR + I + G was selected for the mtSSU and K80 + G for the 5.8S region. The analysis was run using MrBayes (v. 3.2.7a) [58] on the Cipres Science Gateway [59] as described by Olsson et al. [60]. The convergence of the four parallel runs was checked after 1.5 × 10^7^ generations using Tracer (v. 1.5) [61] and graphed using TreeGraph2 (v. 2.15) [62]. 

Based on the insufficient separation between groups within Clade R, a further analysis was run including only the taxa in Clade R, using the mtSSU and complete ITS region, except for the outgroup taxa (*Collema furfuraceum*, *Leptogium caespitosum*, and *L. juressianum*) for which only the mtSSU and 5.8S regions were included. Long and rare insertions present in only a few sequences were removed and also otherwise the same principles followed as in the first analysis. Selected substitution models were HKY + I for mtSSU, GTR + G for ITS1, K80 for 5.8S, and HKY + G for the ITS2 regions; the analysis was run for 10^7^ generations.

Species and Operational Taxonomic Units (OTUs) were defined based on the results of the phylogenetic analyses and morphological and ecological comparisons. Here, OTU refers to a putative species that has not yet been formally described or its species identity cannot be linked to a specific previously known species. Specimens lacking the mtSSU sequence and hence mainly excluded from the first phylogenetic analysis were placed within the clades and OTUs based on the ITS sequences. 

## 3. Results

### 3.1. mtSSU and nuITS Molecular Markers in Leptogium

According to our findings, among *Leptogium* species, the nuITS region works well in species identification and offers more resolution than the mtSSU, which, however, is more useful for the phylogenetic analysis, especially in some groups. Both ITS1 and ITS2 of the nuITS are extremely variable, and the combined length of the ITS1-5.8S-ITS2 regions vary from approximately 480 nucleotides to over 1010 among the studied *Leptogium* species (Figure S1), rendering the regions occasionally unalignable even between closely related taxa. The considerable length and/or variability of the region may also affect the success of PCR and sequencing in some species, and occasionally, mainly for some OTUs in Clades L, N, and Q (Figure S2), full length nuITS regions were not obtained, rendering the length estimations to probable underestimations. Regardless, a clear shift to longer nuITS regions was observed from Clades B–K to Clades L–R (Figure S1). The latter group of clades also formed a monophyletic group in the phylogenetic analysis of mtSSU and 5.8S regions (Figure S2).

### 3.2. Leptogium Morphotypes

**Figure 1:**
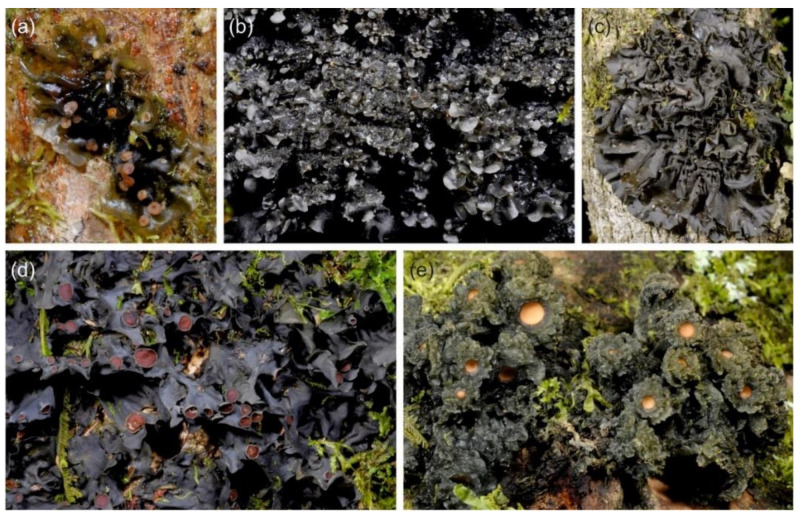
Examples of the *Leptogium* morphotypes from the montane forests of Kenya. (**a**) Fertile morphotype azureum. (**b**,**c**) The morphological variation within morphotype cyanescens include isidiate and phyllidiate species, as well as many transitional forms. (**d**) Morphotype cochleatum with a thick thallus and prominent apothecial margins. (**e**) Morphotype phyllocarpum with abundantly phyllidiate apothecia.

The phylogenetic analysis grouped our *Leptogium* specimens into a number of well-established species and into many putative taxa (Figure S2). Our results also revealed that the described morphologies of many species previously named to occur in the region [32] are shared by more than one phylogenetically distinct OTUs identified in the analyses (Figure S2). Thus, such traditionally used names clearly refer to morphotypes, i.e., potentially diverse assemblages of *Leptogium* taxa that have similar (but not necessarily identical) thallus morphology, and which are not necessarily closely related [63]. We name such assemblages as follows (characteristic morphology in parenthesis): Morphotype adpressum (deeply plicate thallus, usually with sack-like thalline nodules), morphotype austroamericanum (slightly striate thallus surface and mainly laminal isidia), morphotype azureum (thin and smooth thallus lacking symbiotic propagules but often fertile and with inconspicuous apothecial margins; Figure 1a), morphotype brebissonii (olive green to brown thallus and dark laminal isidia), morphotype cochleatum (thick, wrinkled to striate thallus lacking symbiotic propagules, but often fertile and with thick apothecial margins; Figure 1d), morphotype coralloideum (deeply plicate thallus with coralloid isidia), morphotype cyanescens (smooth thallus with isidia and phyllidia on thallus margins and lamina; Figure 1b,c), and morphotype phyllocarpum (deeply plicate thallus, lacking thalline nodules, commonly fertile and with phyllidiate apothecial margins; Figure 1e). 

The most common morphotypes in our material are cyanescens and azureum, represented by approximately 21 and 14 OTUs, respectively (Figure S2). In addition, morphotypes adpressum and cochleatum are relatively common, the first represented by seven OTUs in three main clades and the latter by five OTUs in four main clades (Figure S2). The morphological distinctions between morphotypes, detected OTUs, and species were not always clear-cut. For example, some morphological characters traditionally used to distinguish between different *Leptogium* species, such as thallus striae, or type and position of symbiotic propagules, varied even within single, phylogenetically delimited OTUs. The problem of diffuse and partly shared characteristics was encountered especially between the morphotypes azureum and cyanescens, azureum and cochleatum, austroamericanum and cyanescens, and adpressum and coralloideum.

### 3.3. Leptogium Diversity on the Studied East African Mountains

The phylogenetic analysis of the mtSSU and 5.8S regions divide the *Leptogium* specimens into several distinct clades (Figure S2). Based on the results, the approximately 570 specimens include over 70 established and putative species of *Leptogium*, identified based on the mtSSU and/or nuITS marker regions and often further distinguished by morphology, distribution, and ecology. 

**Figure 2:**
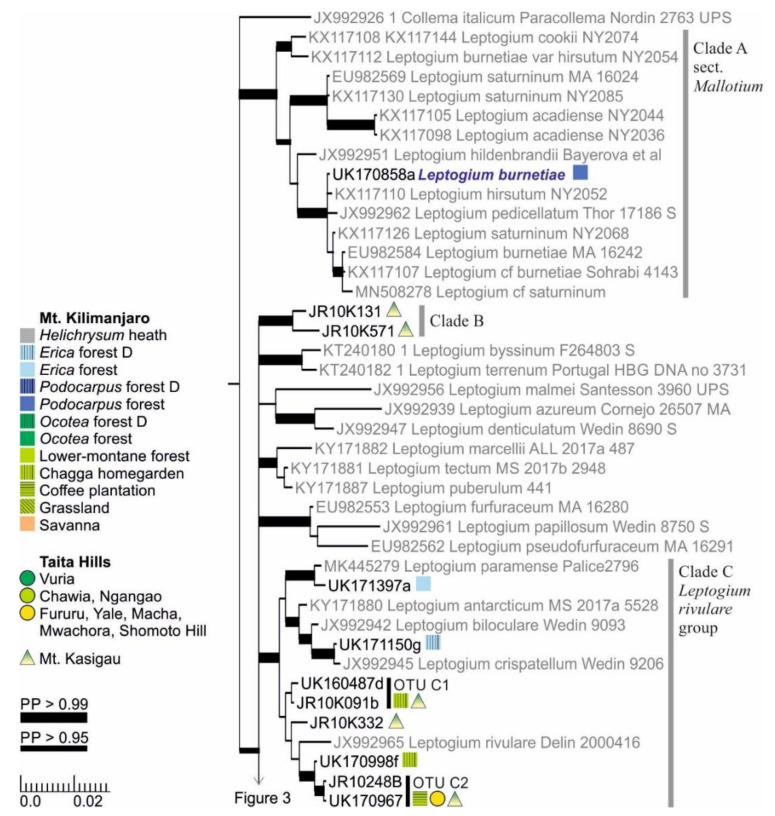
Clades A–C of the Bayesian tree of the genus *Leptogium* based on the mtSSU and 5.8S marker regions (Figure S2). Newly obtained sequences are in black, including one specimen representing each mtSSU variant; all specimens belonging to each clade are listed in Table S1. Established species are in blue. The colored shapes (rectangle, circle, triangle) show the distribution and abundance of the taxa in the studied regions and ecosystem types: On Mt. Kilimanjaro, each vegetation zone is indicated by color while the disturbed ecosystem types are further separated with grid; the widths of the rectangles indicate the number of sample plots in which the taxon was present in each ecosystem type (square = 1). In Taita Hills, each circle indicates presence in one forest fragment. On Mt. Kasigau, each triangle refers to presence on one collection transect, corresponding to the northern, eastern, southern, and western slopes of the mountain. Strong support (>0.95) for a clade (posterior probability, PP) is indicated with a thicker branch while the precise values are shown in Figure S2. The scale refers to nucleotide substitutions per site. D = disturbed.

**Figure 3:**
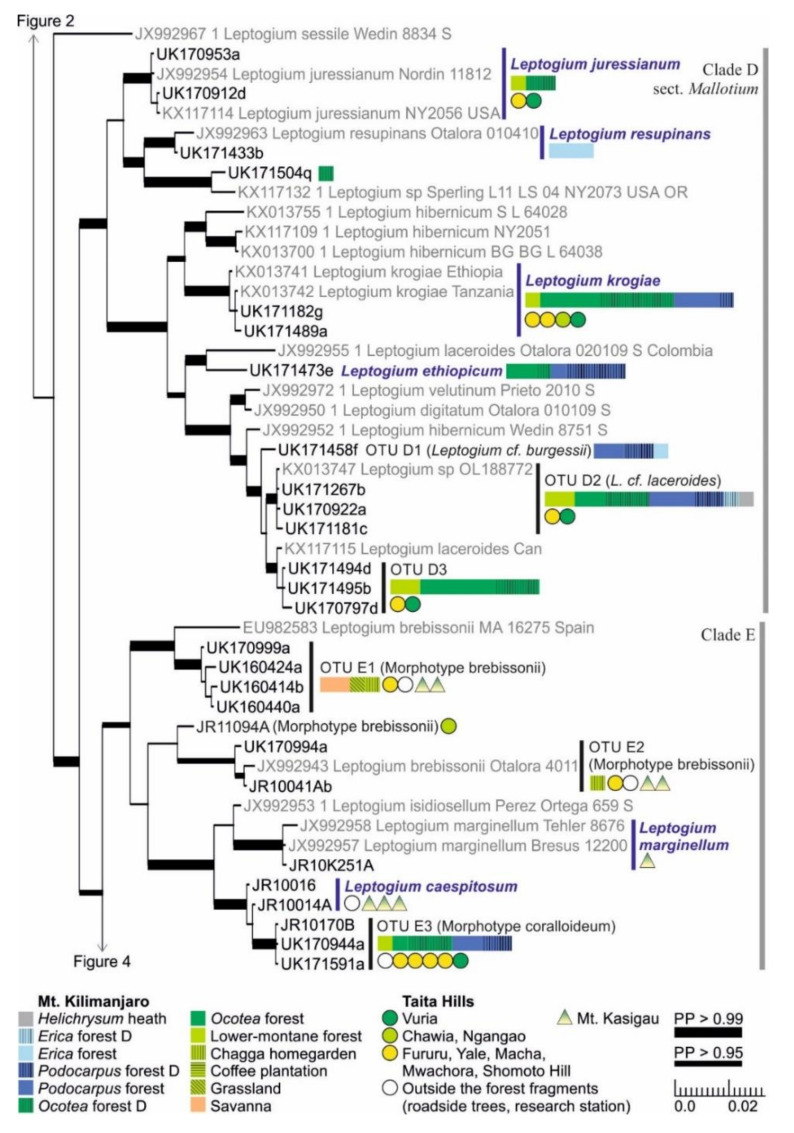
Clades D and E of the Bayesian tree of the genus *Leptogium* based on the mtSSU and 5.8S marker regions (Figure S2). Newly obtained sequences are in black, including one specimen representing each mtSSU variant; all specimens belonging to each clade are listed in Table S1. Established species are in blue. For more detailed explanation, for example, for the colors and shapes indicating the distribution and abundance of taxa, see the caption of Figure 2. D = disturbed; PP = posterior probability (for the precise values, see Figure S2).

The hairy taxa of the section *Mallotium* are separated into several clades, one including the more long-haired species (Clade A) and one with shorter hairs (Clade D) (Figure 2 and Figure 3). Some other species of the section *Mallotium* from other parts of the world are placed between these two major clades. Only one specimen of Clade A, typical *L. burnetiae* characterized by long white hairs on the lower side and dark isidia on the upper side, was found from *Podocarpus* forest (2700 m alt.) on Mt. Kilimanjaro. Clade B includes two specimens with adnate, relatively smoot, and nonhairy thalli with abundant symbiotic propagules (phyllidia and/or isidia), both collected from montane forest on Mt. Kasigau (Figure 2). Clade C, here named the *Leptogium rivulare* group, includes several OTUs, among others, with some resemblance to the species *L. rivulare* but with symbiotic propagules and not exhibiting the characteristic, periodically inundated ecology of *L. rivulare* (Figure 2). Most of these specimens were collected from relatively dry and open lower-elevation forests of Mt. Kasigau or from homegardens and coffee plantations of Mt. Kilimanjaro. Finally, Clade C also includes two *Leptogium* specimens collected from *Erica* forest (>3500 m) on Mt. Kilimanjaro, one related to *Leptogium paramense* and the other one to *L. crispatellum*. 

The short-haired taxa of section *Mallotium*, including several classical species, were further divided into two clades (Clade D; Figure 3). The first group includes *Leptogium juressianum*, *L. resupinans*, and specimen UK171504q, with hairs composed of cylindrical cells. *L. resupinans*, with velvety tomentum covering the upper surface and otherwise smooth thalline exciple, is rare on the study area and was only collected from the high-altitude *Erica* forest on Mt. Kilimanjaro. *L. juressianum*, with felt-like hair and marginal isidia, was collected from the lower-montane and *Ocotea* forests of both Mt. Kilimanjaro and Taita Hills. In addition, one specimen with *L. juressianum*-like morphology (UK171504q) from the same habitat formed a separate clade with a specimen from the USA. The second group within Clade D includes the remaining taxa, all having hairs composed of spherical cells. *Leptogium krogiae*, which has a striate upper surface and laminal isidia, and usually also coarse tufts of hairs mainly on the lower surface, is a common species in the forests of Mt. Kilimanjaro and Taita Hills. Another common species, *Leptogium* OTU D2, from the montane forest zones of Mt. Kilimanjaro resembles *L. laceroides* in having marginal isidia and tufts of hair mainly on the lower side. However, these specimens are placed in a clade separate from specimens of *L. laceroides* previously sequenced from Colombia and Canada. The second subgroup of Clade D also includes three commonly fertile species with hairs mainly on the lower surface and mainly collected from forests of Mt. Kilimanjaro: *Leptogium ethiopicum*, with isidiate or nodulous apothecial margins, common in the *Ocotea* and especially in the *Podocarpus* forest; *Leptogium* OTU D1 from the high elevation *Podocarpus* and *Erica* forests, resembling *L. burgessii* with abundantly and clearly phyllidiate apothecial margins; and OTU D3, with specimens with phyllidiate to nodulous apothecial margins. 

Clade E (Figure 3) is comprised of *Leptogium* species with deeply plicate thalli. The specimens from East Africa representing the morphotype brebissonii are divided into three distinct OTUs, and most of the specimens have been collected from lower elevations, from open habitats, including woodlands, savanna, and gardens. *Leptogium marginellum*, with small marginal and isidiate to phyllidiate apothecia, was collected only once, from the transition zone between semievergreen and evergreen forest on Mt. Kasigau (1100 m alt.). In addition, *Leptogium caespitosum*, characterized by apothecia with nodulous margins, prefers similar habitats on Mt. Kasigau. Although the specimens of *Leptogium caespitosum* did not form a monophyletic clade in the analysis of mtSSU and 5.8S regions, the species is supported by several characters within the complete nuITS region. *Leptogium* OTU E3 of the morphotype coralloideum is the most common species in Clade E in the study area and was found from forests of both Mt. Kilimanjaro and Taita Hills.

**Figure 4:**
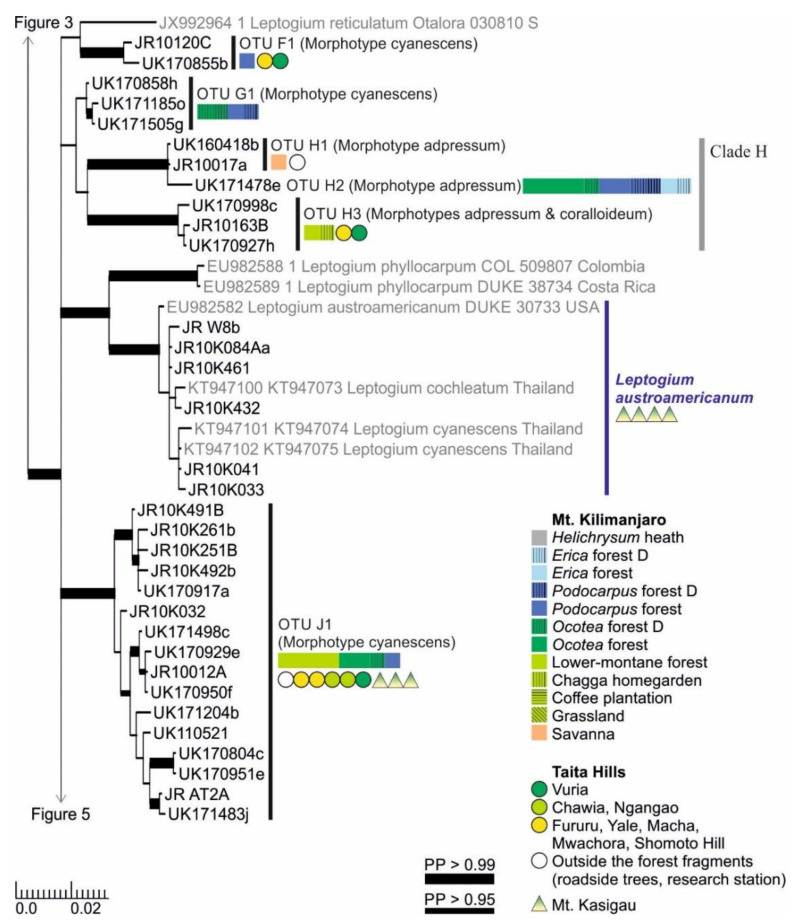
Clades F–J of the Bayesian tree of the genus *Leptogium* based on the mtSSU and 5.8S marker regions (Figure S2). Newly obtained sequences are in black, including one specimen representing each mtSSU variant; all specimens belonging to each clade are listed in Table S1. Established species are in blue. For more detailed explanation, for example, for the colors and shapes indicating the distribution and abundance of taxa, see the caption of Figure 2. D = disturbed; PP = posterior probability (for the precise values, see Figure S2).

Clades F and G both include only one OTU, both belonging to the morphotype cyanescens (Figure 4). Clade H includes three OTUs, all with deeply plicate and often abundantly nodulous thalli of the morphotype adpressum (Figure 4). *Leptogium* OTUs H1 and H2 are not well separated in the phylogenic tree, but their habitats are quite different: H1 has only been found from relatively low elevations in savanna and garden habitats, while H2 is relatively common in the *Ocotea*, *Podocarpus*, and *Erica* forests of Mt. Kilimanjaro. The clearly distinct third species of this clade, OTU H3, has been mainly collected from lower-montane forests. The specimens are usually quite nodulous representatives of the morphotype adpressum, but occasionally the nodules turn to more isidium-like structures typical for the morphotype coralloideum.

*Leptogium austroamericanum* is common in low-elevation woodlands of Mt. Kasigau (< 1200 m alt.) but has not been found from the other mountains (Figure 4). It groups together with *Leptogium austroamericanum* from the USA and specimens identified as *L. cochleatum* and *L. cyanescens* from Thailand. Specimens of Clade J are combined under one OTU, common in the montane forests of all studied mountains (Figure 4). They all belong to the morphotype cyanescens but include some morphological and phylogenetic variation, but clear links between the subtle morphological variation and phylogenetically supported subgroups has not yet been identified.

**Figure 5:**
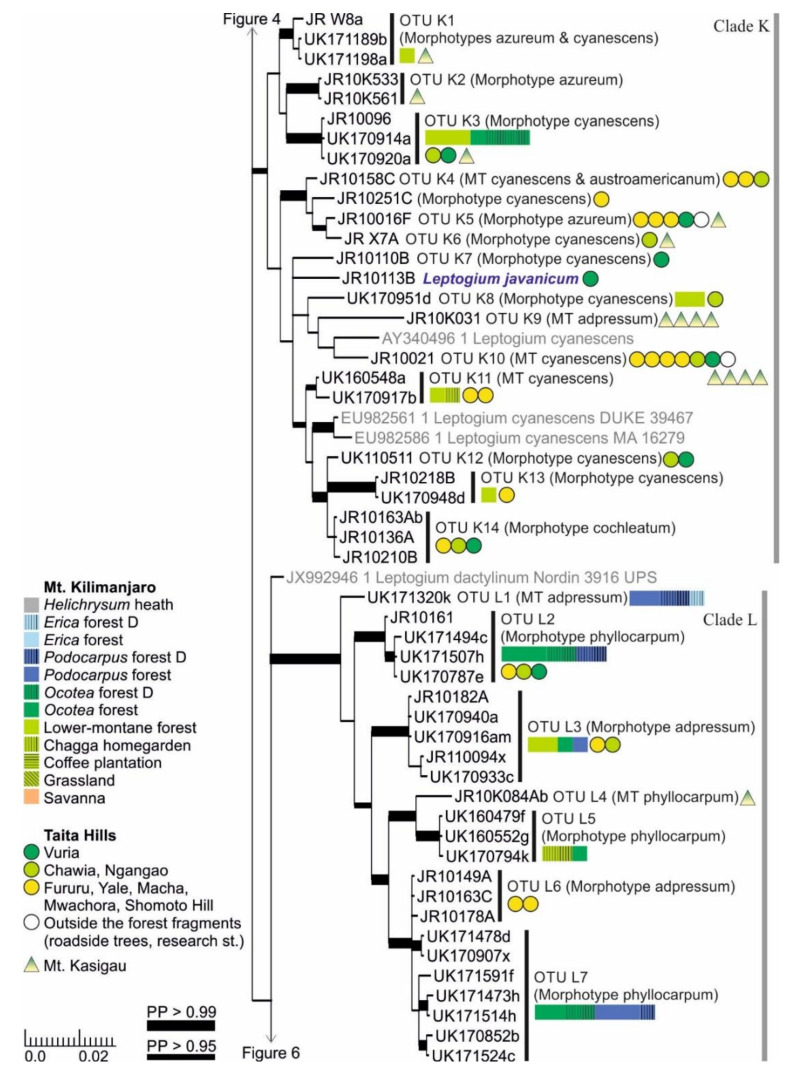
Clades K and L of the Bayesian tree of the genus *Leptogium* based on the mtSSU and 5.8S marker regions (Figure S2). Newly obtained sequences are in black, including one specimen representing each mtSSU variant; all specimens belonging to each clade are listed in Table S1. Established species are in blue. For more detailed explanation, for example, for the colors and shapes indicating the distribution and abundance of taxa, see the caption of Figure 2. MT = Morphotype(s); D = disturbed; PP = posterior probability (for the precise values, see Figure S2).

Clade K includes a number of *Leptogium* species from the lower-montane forest zone of the studied mountains (Figure 5). The OTUs mainly belong to the common morphotypes azureum and cyanescens and, occasionally, both morphotypes are present in one OTU. The clade also includes *Leptogium javanicum*, a species with conspicuous pedicellate apothecia, only collected from a restricted area near the highest summit of the Taita Hills. Another deviant from the general theme is represented by *Leptogium* OTU K9 with specimens mainly of the morphotype adpressum. *Leptogium* OTU K9 is common in the low-elevation woodland of Mt. Kasigau but has not been collected from the other mountains. *Leptogium* OTU K14 with a thick and rugose thallus of the morphotype cochleatum has only been collected from some forest fragments in the Taita Hills.

Clade L includes seven putative species of the morphotypes adpressum and phyllocarpum (Figure 5). However, previously sequenced *Leptogium phyllocarpum* sequences in GenBank from Colombia and Costa Rica do not fall into this clade but form a sister group to *L. austroamericanum* (Figure 4). *Leptogium* OTUs L6 and L7 are not well separated in the phylogenetic analysis, but their thallus morphology and habitats are quite different, with the former taxon representing the morphotype adpressum and so far only collected from the forests of Taita Hills, and the latter being of the morphotype phyllocarpum and so far only known from *Ocotea* and *Podocarpus* forests of Mt. Kilimanjaro. 

**Figure 6:**
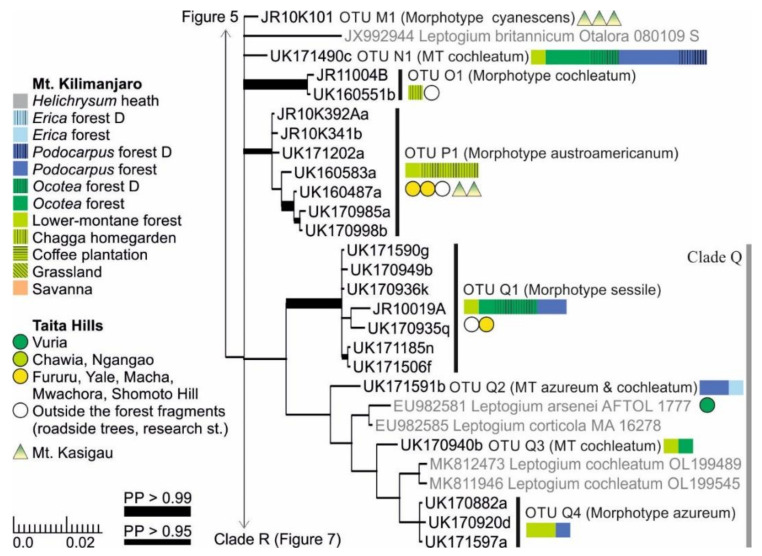
Clades M–Q of the Bayesian tree of the genus *Leptogium* based on the mtSSU and 5.8S marker regions (Figure S2). Newly obtained sequences are in black, including one specimen representing each mtSSU variant; all specimens belonging to each clade are listed in Table S1. For more detailed explanation, for example, for the colors and shapes indicating the distribution and abundance of taxa, see the caption of Figure 2. MT = Morphotype(s); D = disturbed; PP = posterior probability (for the precise values, see Figure S2).

Clades M, N, and P all include only one *Leptogium* OTU each (Figure 6). *Leptogium* OTU M1 grows in the lower-elevation woodlands of Mt. Kasigau and represents the morphotype cyanescens. *Leptogium* OTU N1, which is common in the *Ocotea* and *Podocarpus* forests of Mt. Kilimanjaro, has a thick thallus and robust apothecia of the morphotype cochleatum. *Leptogium* OTU O1, which has only been collected from disturbed environments, has the striate apothecial margins of the morphotype cochleatum, but it has thinner and smoother thallus lobes than OTU N1. *Leptogium* OTU P1 is common in the lower-montane forests of all three mountains and closely resembles *L. austroamericanum*, with which it occasionally grows together on Mt. Kasigau.

Clade Q includes several relatively robust and commonly fertile *Leptogium* species (Figure 6). *Leptogium* OTU Q1 mainly resembles *L. sessile* in having a thick striate to plicate thallus and initially immersed apothecia with thick margins. However, the representative of *Leptogium sessile* in GenBank from Argentina does not fall within this group but is situated between the *L. rivulare* group and the short-haired *Mallotium* group (Clades C and D; Figure S2). *Leptogium* OTUs Q2–Q4 represent the morphotypes azureum and cochleatum and also group together with two *L. cochleatum* sequences from Norway. The poor support in this part of the tree is obviously caused by the lack of overlapping marker regions: the GenBank accessions of *Leptogium arsenei* and *L. corticola* only include mtSSU sequences and that of *L. cochleatum* only nuITS sequences.

**Figure 7:**
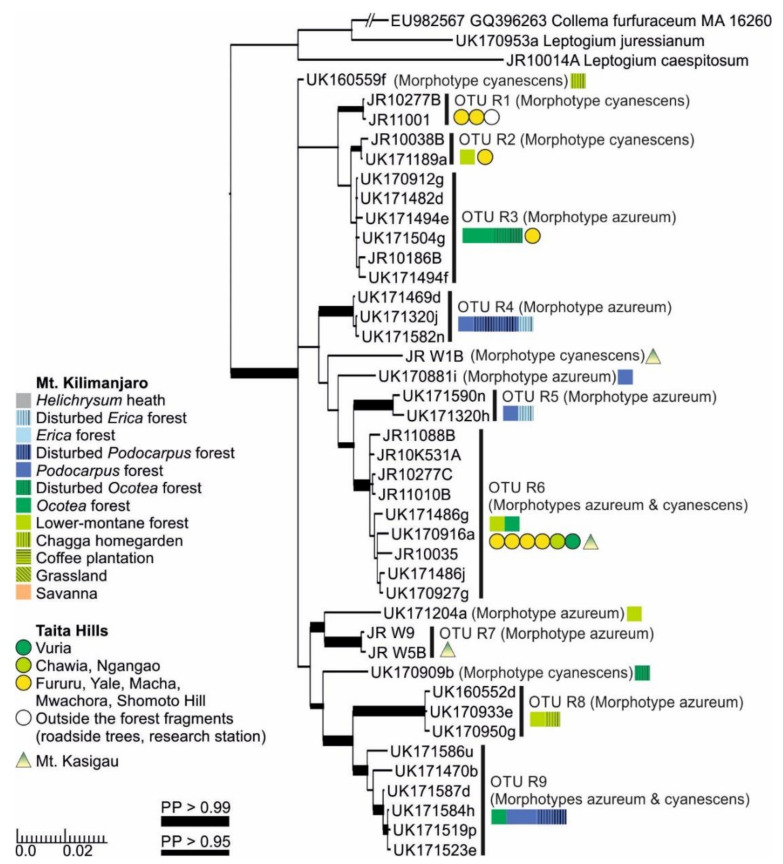
Bayesian tree of the genus *Leptogium* Clade R (Figure S2) based on the mtSSU and nuITS marker regions. All specimens belonging to each clade are listed in Table S1. For more detailed explanation, for example, for the colors and shapes indicating the distribution and abundance of taxa, see the caption of Figure 2. PP = posterior probability.

The separate analysis including the complete mtSSU and nuITS regions for Clade R (Figure 7) provided some additional clarity and support in comparison to the analysis based on only the mtSSU and 5.8S regions (Figure S2). The clade is now split into 14 OTUs that correspond more closely with ecological than morphological differences. All the OTUs represent the morphotypes azureum and cyanescens, or occasionally both. For example, within *Leptogium* OTU R6, even though phylogenetically mixed, all specimens from Mt. Kilimanjaro are fertile and without symbiotic diaspores, while most specimens from Taita Hills have isidia or phyllidia. As a whole, however, the full range of morphological variation within the clade is still insufficiently understood. While most OTUs within this clade appear to prefer lower-montane forests, some, for example OTUs R4, R5, and R9, have only been collected from the high-elevation *Podocarpus* and *Erica* forests on Mt. Kilimanjaro.

### 3.4. Diversity and Ecosystems

**Figure 8:**
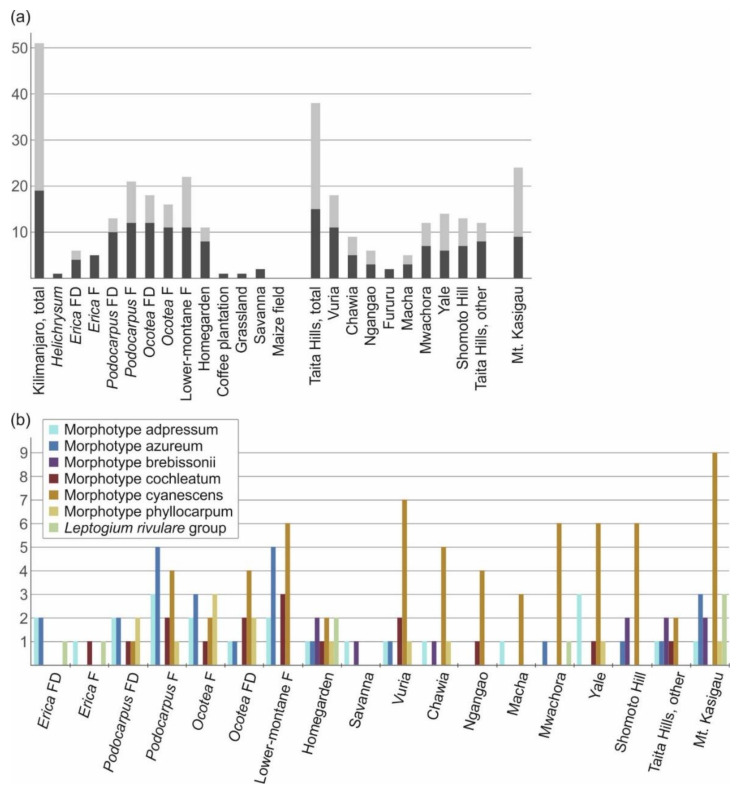
*Leptogium* diversity in different habitats of East African mountains, represented by different ecosystem types on Mt. Kilimanjaro, different forest fragments in the Taita Hills, and Mt. Kasigau as a whole. (**a**) Total species diversity of *Leptogium* in each habitat type (dark grey = proportion of classical, previously known species; light grey = proportion of new diversity discovered in this study). (**b**) Distribution of the common *Leptogium* morphotypes among the habitat types (including habitats with at least three different morphotypes). F = forest; D = disturbed habitat.

Of the three montane regions studied, the highest diversity of putative *Leptogium* species was found on Mt. Kilimanjaro, with 51 species (Figure 8a). Regarding the different ecosystem types on Mt. Kilimanjaro, lower-montane forest has the highest number of species (22), followed by natural *Podocarpus* forest (21), disturbed *Ocotea* forest (18), *Ocotea* forest (16), and disturbed *Podocarpus* forest (13). Of the more altered ecosystems, the Chagga homegardens supported 11 different *Leptogium* species, however, the species number drastically declines with the intensifying disturbance of coffee plantations, grasslands and maize fields. In the Taita Hills, we detected 38 species of *Leptogium*, with the highest diversity (18 species) in the relatively small fragment of montane forest on Vuria; more than ten *Leptogium* species were also collected from Mwachora, Yale, and, somewhat surprisingly, from a tiny forest remnant on Shomoto Hill. A total of 24 *Leptogium* species were collected from Mt. Kasigau. The most common *Leptogium* morphotype in the studied ecosystems was cyanescens and it is particularly common in the low elevation forests and disturbed habitats, especially in the Taita Hills (Figure 8b). In addition, the proportion of typically fertile species seems to correlate with the elevation, being 58–67% in the *Erica* forest, both types of *Podocarpus* forests and *Ocotea* forest, 45–47% in the disturbed *Ocotea* and lower-montane forest, 35% at the highest peak of Taita Hills (Vuria), and considerably less in all the other forest fragments in the Taita Hills and the habitat types at lower elevations.

## 4. Discussion

We discovered over 70 putative *Leptogium* species from three mountain regions in Kenya and Tanzania, including nine established species previously known from the area and over 60 phylogenetically, morphologically, and/or ecologically defined OTUs, of which some may also represent established species. However, since the traditionally used defining morphological characters are often shared by several putative species and no DNA data from other parts of the world is available for comparison, more definite statements regarding the species identity of these taxa cannot currently be made. A summary of our findings and the *Leptogium* species and morphotypes in East Africa is provided in Table 1. 

**Figure 9:**
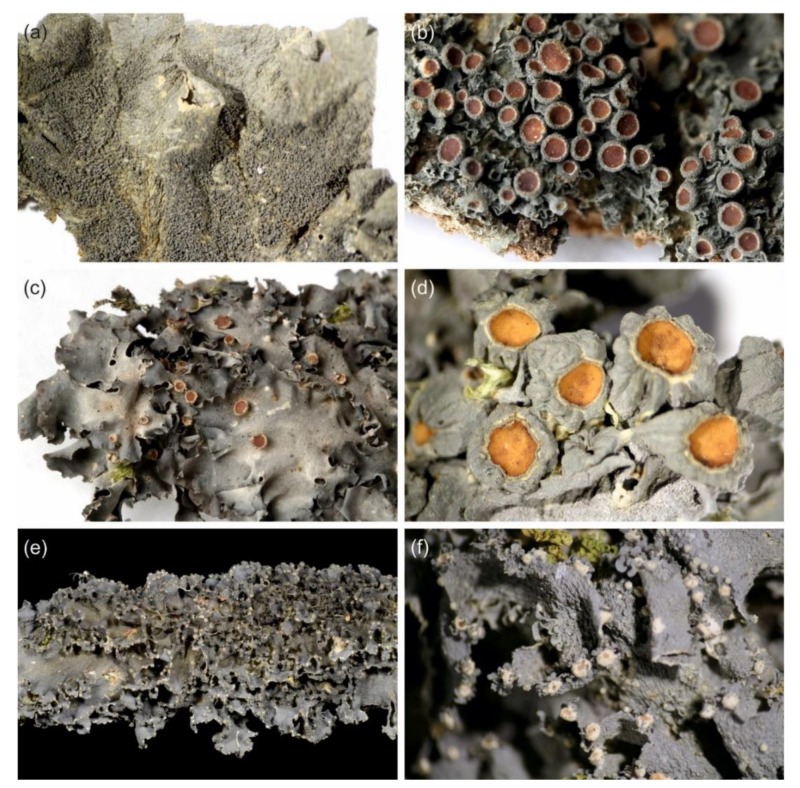
*Leptogium* species from East Africa. (**a**) Abundantly isidiate and striate to slightly wrinkled thallus of *Leptogium austroamericanum* (JR10K461). (**b**) Abundantly fertile *Leptogium caespitosum* with nodular apothecial margins (JR10K401A). (**c**) *Leptogium ethiopicum* with isidiate apothecial margins (UK171584f). (**d**) Pedicellate apothecia of *Leptogium javanicum* (JR10119B). (**e**) *Leptogium marginellum* imaged in situ on a branch. (**f**) The small, phyllidiate, marginal apothecia and wrinkled surface of *Leptogium marginellum* (JR10K251A).

Most of the observed taxa were clearly identified in the phylogenetic analysis of the mtSSU and 5.8S marker regions and supported by morphology and ecology and further comparisons of the complete nuITS region. Of the 25 taxa previously reported from East Africa [32,33,36], we found nine that could be relatively unambiguously identified: *Leptogium austroamericanum* (Figure 9a), *L. burnetiae, L. caespitosum* (Figure 9b), *L. ethiopicum* (Figure 9c), *L. javanicum* (Figure 9d), *L. juressianum, L. krogiae, L. marginellum* (Figure 9e,f), and *L. resupinans*. To our knowledge, *Leptogium ethiopicum, L. juressianum*, and *L. resupinans* are new reports for Tanzania, and *L. caespitosum*, *L. ethiopicum*, and *L. javanicum* have not previously been represented by voucher sequences in public databases. Additionally, *Leptogium burgessii* is very likely present in our material and represented by either OTU D1 or D3, and it is possible that some of the OTUs of the morphotypes adpressum, azureum, brebissonii, and coralloideum also include the name species.

The identification of *Leptogium austroamericanum, L. juressianum*, *L. krogiae*, *L. marginellum*, and *L. resupinans* is supported by the phylogenetic analysis and specimens from other parts of the world included in the analysis. The East African specimens of *Leptogium austroamericanum*, species originally described from Central America [64], fits the morphological description of the species and is placed within the same clade with *L. austroamericanum* from the USA (Figure 4). The clade also includes specimens identified as *Leptogium cochleatum* and *L. cyanescens* from Thailand, mixed with the *L. austroamericanum* specimens from East Africa. However, since there are no significant morphological, ecological, or distributional differences between different East African specimens, or phylogenetic support for further divisions within the clade, all the specimens are, for now, placed under *Leptogium austroamericanum*. Morphologically, this species may be confused especially with *Leptogium* OTU P1, which shares some of the habitats with *L. austroamericanum* but has a wider distribution in the lower-montane forests on all the studied mountains. In addition, Swinscow and Krog [32] acknowledged the presence of a possible second species very similar to *Leptogium austroamericanum* but differing in some apothecial characters.

*Leptogium juressianum*, originally described from Portugal [65], occurs on Mt. Kilimanjaro and Taita Hills in the lower-montane and *Ocotea* forests (1700–2400 m alt.), which is somewhat lower than previously reported [32]. The species can be confused with another species, sharing the same habitat and a similar morphology, represented in our material by only a single specimen (Figure 3). According to Kitaura and Marcelli [66], *Leptogium juressianum* may represent a species complex and needs to be re-evaluated—our results seem to confirm this.

*Leptogium krogiae* was recently described from Tanzania [36], and we can confirm that this species is common in the montane forests of East Africa. *Leptogium marginellum* was originally described from the Caribbean [67] and is also known from Cuba and Ecuador [29]. The species has been previously reported from mountain rainforest and open woodland in Kenya, Tanzania, and Uganda [32,33], but we found only one specimen from a montane forest on Mt. Kasigau. 

*Leptogium resupinans*, originally described from Bolivia [68], is also known from the Canary Islands [29]. We only found the species from undisturbed *Erica* forests on Mt. Kilimanjaro, which resembles the habitat of the type specimen [32].

*Leptogium burnetiae*, represented in our material by a single specimen collected from a *Podocarpus* forest on Mt. Kilimanjaro (2700 m), well fits the description of the species, originally found from East Africa [69] and the phylogenetic analysis grouped it with other *Leptogium burnetiae* specimens from different parts of the world (Figure 2).

*Leptogium caespitosum* was originally described from South Africa [70] and is easy to identify on the basis of its characteristic morphology, especially when fertile. It has previously been reported to be widespread but not common in East Africa [32]. We mainly collected this species from Mt. Kasigau, but it is quite likely to also occur in comparable lower-elevation woodland habitats on the other mountains. 

*Leptogium ethiopicum*, originally described from a high-elevation (> 3000 m) Ethiopian montane forest has been previously reported from Kenya [69], and now we found it from high-elevation montane forests on Mt. Kilimanjaro. The species could be confused with *Leptogium* OTU D3 which, however, seems to prefer lower elevations, including some forest fragments in Taita Hills.

*Leptogium javanicum*, a species originally described from Java [71], was only found from a fragment of montane mist forest near the summit of Vuria, where the species appears to be locally common. Previously it has been reported from a hand full of locations in Kenya and Tanzania [32,33]. The species has also been reported from Brazil [72], but a comparison with the published description suggests that the Brazilian taxon is not conspecific with our material from Kenya. This supports the interpretation [72] that *Leptogium javanicum* probably represents a species complex.

In addition to the taxa discussed above, we confirmed the existence of a plethora of other, currently undescribed putative *Leptogium* species, some of which morphologically greatly resemble previously described taxa. This points towards widespread morphological homoplasy in the genus *Leptogium*, i.e., the presence of similar morphologies among phylogenetically unrelated taxa. This emphasizes the general need for using DNA markers when assessing the diversity of *Leptogium* species, especially in regions where the diversity has been poorly studied.

Prime examples of morphological homoplasy include the morphotypes adpressum and coralloideum, both including OTUs identified in this study. Both *Leptogium adpressum* and *L. coralloideum* have been previously reported from East Africa [32,33] but not originally described from the region [73] and no reference DNA data is available for either species in GenBank. In our material, *Leptogium* species that share the similar morphology, i.e., grey, deeply plicate thallus either with thalline nodules or isidia, are present in several different clades. Furthermore, the difference between the specimens representing the two morphotypes may sometimes be far from clear-cut. The morphotypes represent at least eight putative species, the morphotype adpressum being clearly more species rich and morphologically diverse (Figure S2). Recent descriptions of *Leptogium coralloideum* from Brazil [72,74], the type location, suggest that *Leptogium* OTU E3 might not be *Leptogium coralloideum*, but one of the other taxa mentioned above might be. Previous reports form East Africa have mentioned *Leptogium coralloideum* to be common from sea level to high elevations (>3000 m) and *L. adpressum* to occur at elevations between 1000 and 3400 m [32,33]. Clearly, both statements are oversimplifications of a complex matter. 

In addition, *Leptogium burgessii* is a morphological taxon that had not been previously sequenced and includes more than one possible phylogenetic species in our study area. The two species in our material include *Leptogium* OTU D1 from the high-montane *Podocarpus* and *Erica* forests on Mt. Kilimanjaro and OTU D3 from lower-montane and *Ocotea* forests of Mt. Kilimanjaro and from the Taita Hills (Figure 3). Previously, *Leptogium burgessii* has been reported from shady montane forests and the ericaceous zone (1900–3500 m) in East Africa [32,35]. These reports have probably been based on the occurrences of both species we now detected, and possibly also *L. ethiopicum*, which has been treated as a synonym of *L. burgessii* [37]. 

*Leptogium brebissonii*, originally described from the Canary Islands [75], is comprised of at least four closely related but distinct species, of which three are present in East Africa (Figure 3). The nonmonophyly of *L. brebissonii* has also been pointed out in a previous study [29]. Based on sequences from the GenBank, one of the East African species also occurs in Colombia, while the species from Spain was not detected in our region. Previously, *Leptogium brebissonii* has been reported to occur in shady montane forests (1100–2500 m) in Ethiopia, Kenya, and Uganda [32]. Our observations are from slightly lower elevations in savanna, low-elevation woodlands, and lower-montane forest habitats and also include several sampling locations in Tanzania. 

*Leptogium laceroides*, *L. sessile*, *L. cochleatum*, and *L. phyllocarpum* are all species that have been previously reported from East Africa [32,33,34,35], but the specimens collected by us do not correspond with published sequences of these species from other parts of the world. The East African specimens resembling *Leptogium laceroides* and *L. sessile*, both described from Central America [76,77], represent lineages of their own, but were not grouped together with specimens from Colombia and Argentina (Figure 2 and Figure 5). This suggests that the East African specimens actually represent separate species. Previously, *Leptogium laceroides* has been reported in East Africa from lower to high elevation montane forests (1500–3400 m) and *L. sessile* from lower to middle montane habitats (1100–2500 m) [32,33]. In our material, the *L. laceroides*-like OTU D2 was very common on Mt. Kilimanjaro from the lower-montane forest to very high elevations and was the only *Leptogium* species found growing in the alpine *Helichrysum* heath. The *L. sessile*-like OTU Q1 had a more restricted distribution. 

In addition, specimens corresponding morphologically with *Leptogium phyllocarpum*, originally described from Brazil [78], and *Leptogium cochleatum*, originally described from England [79], were split into several distinct taxa in the phylogenetic analysis. The specimens of morphotype phyllocarpum were divided into four different species, all within Clade L, and having distributions from lower-montane forests to upper-montane *Podocarpus* forests. In previous literature, *L. phyllocarpum* has been reported to be common in gardens, woodlands, and forests of East African mountains [32,33]. Specimens of morphotype cochleatum were present in several different clades, including Clade Q, which also included *Leptogium cochleatum* specimens previously sequenced from Norway (Figure 6). We mainly delimited the morphotype cochleatum to specimens with conspicuously thick and rugose thalli and thick apothecial margins, corresponding to the species as portrayed by Swinscow and Krog [32]. Some such specimens came quite close to the *L. sessile*-like thalli of *Leptogium* OTU Q1. In the other end of the morphological continuum there were specimens with striate but relatively thin thalli, somewhat closer to the *Leptogium cochleatum* described from Europe [79]. Previous literature reports *Leptogium cochleatum* as being fairly common in the montane forests of East Africa [32,35]. We can confirm that especially *Leptogium* OTU N1 and some other species of this complex are common in some forest zones while others are uncommon, like *Leptogium* OTU O1, which was only collected from relatively open habitats. 

The extreme cases of the morphological homoplasy include the morphotypes azureum and cyanescens. Both thallus morphologies were very common in our East African material and were represented by some OTUs of almost all nonhairy clades (Figure S2). The separation of these morphotypes from others was not always easy and, on several occasions, specimens representing several morphotypes were present within one OTU, especially within Clade R (Figure 7). The main differences between a *L. cyanescens*-like and a *L. azureum*-like morphology has classically been that *L. cyanescens* bears isidia and/or phyllidia, while *L. azureum* should have neither but tends to be commonly fertile. Our results clearly show that this morphological dichotomy is not always indicative of phylogenetic separation in *Leptogium*. In general, the morphotype cyanescens is more common in lower-montane forests and especially in the Taita Hills, where it is by far the most common and abundant *Leptogium* morphotype, while the morphotype azureum is more prevalent at higher elevations and on Mt. Kilimanjaro. *Leptogium* OTU R6 is an especially interesting case, as the specimens of this OTU collected from the Taita Hills produce symbiotic propagules, while specimens of the same species from Kilimanjaro do not but are commonly fertile!

We did not collect *Leptogium asiaticum, L. digitatum, L. furfuraceum, L. punctulatum*, *L. rivulare*, *L. vesiculosum*, or morphologically similar taxa from our study area, even though all these have been previously reported from East Africa [32,33,35]. Our specimens included several specimens of the *Leptogium rivulare* group (Figure 2) which, however, did not group together with *L. rivulare* in the phylogenetic analysis, lack apothecia but often have isidia, and have a different ecology than *L. rivulare*. Some of the specimens also bear structures resembling the (semi)marginal globose pycnidia described from *L. rivulare* in Tanzania [33]. *Leptogium asiaticum, L. digitatum,* and *L. furfuraceum* are all hairy *Leptogium* species reported to be uncommon or rare in the montane forests of Kenya and/or Tanzania [32]. *L. punctulatum*, characterized by two-layered and folded thallus lobes and attachment pits, has been reported from a single location in Uganda and *L. vesiculosum*, with conspicuous pedicellate apothecia, is also only known from two locations in East Africa [32]. 

Of the over 70 *Leptogium* species and OTUs identified, 23 were only collected from either Mt. Kasigau, a single forest fragment in Taita Hills, or one ecosystem type on Mt. Kilimanjaro. Some of these taxa were collected only once, but the group also included a number of locally common species. This type of distribution was especially characteristic of Mt. Kasigau, which had eight *Leptogium* species that have so far not been collected from other sites. These include, for example, the locally common *Leptogium austroamericanum* and OTU K9. Another interesting example is *Leptogium* OTU K10, which was by far the most common *Leptogium* species in the woodlands and lower-montane forests on Taita Hills and Mt. Kasigau but seemed to be absent from Mt. Kilimanjaro. One possible reason for this absence could be the lack of suitable habitats: extensive degradation of low elevation forests and woodlands and conversion to agricultural land [5,17] may have largely destroyed its primary habitats. 

As a whole, most observed differences in *Leptogium* species composition between the different mountains can be explained by differences in the ecosystem types present. Mt. Kilimanjaro has the most extensive environmental gradient ranging from savanna to alpine heath and also the consequent highest number of observed species. The relatively high number of species of Taita Hills in comparison to Mt. Kasigau is probably mainly due to the more excessive, albeit now badly fragmented and partly deteriorated, total area of lower-montane forest in the Taita Hills. The higher proportion of mainly fertile species at the higher elevations, or the higher proportion of symbiotically dispersing species at lower elevations is an interesting finding, which requires further study. Could speciation have been faster in *Leptogium* species that reproduce mainly via asexual symbiotic diaspores? Could such a phenomenon have been especially pronounced in lower-montane forests with a wide range of available niches? Some lichenized fungi may also expand their ecological tolerance by partnering with variable photobionts [80], and, for species specialized in ecosystems farther from the environmental optimum, distribution via fungal spores may enable them to more often partner with photobionts specialized to the exact conditions of the new habitat.

Our results reveal that the diversity of *Leptogium* in East Africa is much higher than previously thought, and that the number of species there may exceed what has so far been reported for any other region of the world. The montane forests of East Africa are widely known for their unique biodiversity, and our findings demonstrate that this reputation also holds true for the genus *Leptogium*. Local endemism may be high, but the poor sampling of other mountain regions and tropics in general, currently prevents definite conclusions. Many of the classical species previously reported from our study area clearly represent morphotypes, i.e., groups of morphologically similar species that are not necessarily closely related or more closely related species complexes. A similar situation has also been observed in many other lichen groups in studies utilizing DNA markers and phylogenetic analysis [81,82,83], highlighting that some thallus features commonly used in species identification do not always reflect phylogeny and that we still have a poor understanding of factors that affect the development of a lichen phenotype [84,85,86]. Our results also highlight the perhaps insurmountable challenge of rigorously dealing with intraspecific variability, interspecific overlap, and very limited number of morphological and anatomical characters available for the accurate identification of *Leptogium* species. They also underline the urgent need for detailed molecular studies of fresh material collected from the type localities of many traditionally delimited *Leptogium* species, which have so far been thought to have almost cosmopolitan distributions.

## 5. Conclusions

Species diversity in the genus *Leptogium* is much higher than previously known, especially in East Africa, but probably also in other parts of the world. Many species as traditionally circumscribed represent species groups and the morphological characters used to distinguish between taxa require critical review. At present, reliable estimation of global species diversity and distribution patterns is prevented by insufficient taxon sampling and the lack of molecular data especially from tropical mountain areas, which can be expected to harbor most of the diversity in the genus.

## Figures and Tables

**Table 1:** Diversity of the genus *Leptogium* in the study area. The distribution in Kenya (K) and Tanzania (T) also includes previous literature reports (Refs); if more than one morphologically similar taxa are present in the region the previous reports are listed after *Leptogium* morphotypes. Ecology is reported as observed in this study.

***Leptogium* Species**	**Distribution**	**Ecology**		**Comments**	**Refs**
*L. austroamericanum*	K	Lower-elevation woodland, common.			
*L. burnetiae*	K, T	High-montane *Podocarpus* forest, rare.			[32,33,34,35]
*L. caespitosum*	K, T	Lower-montane forest/woodland, common.			[32]
*L. ethiopicum*	K, T ^1^	Montane *Podocarpus* and *Ocotea* forest, common.			[64]
*L. javanicum*	K, T	Montane forest, rare.			[32,33]
*L. juressianum*	K, T ^1^	Lower-montane and montane forest.			
*L. krogiae* ^2^	K, T	Montane forest, common.			[32,33,36]
*L. marginellum*	K, T	Lower-montane forest/woodland, rare.			[32,33]
*L. resupinans*	K, T ^1^	High-montane *Erica* forest.			[32,34]
***Leptogium* morphotypes**		**Putative species**	**Clade(s)**		
Adpressum	K, T	6	H, K, L		[32,34]
Austroamericanum	K, T	~2	I, (K), P	Name species occurs in East Africa.	[32,33,35]
Azureum	K, T	~14	K, Q, R		[32,33,35]
Brebissonii	K, T ^1^	3	E		[32]
Burgessii	K, T	1–2	D	Name species probably occurs in East Africa.	[32,35]
Cochleatum	K, T	4–5	K, N, O, Q	Name species probably does not occur in East Africa.	[32,33,35]
Coralloideum	K, T	~1	E, (H)		[32,33,34]
Cyanescens	K, T	~21	F, G, J, K, M, R		[32,33,35]
Juressianum	K, T	2	D	Name species occurs in East Africa.	[32]
Laceroides	K, T	1	D	Name species probably does not occur in East Africa.	[32,33,34]
Phyllocarpum	K, T	4	L	Name species probably does not occur in East Africa.	[32,33]
Sessile	K, T	~1	Q	Name species probably does not occur in East Africa.	[32,33]
**Other groups**					
Clade B	K	1–2	B		
*L. rivulare* group	K ^1^, T	6	C		[33]
***Leptogium* species previously reported from East Africa but not found in this study**					
*L. asiaticum*	K, T				[32]
*L. digitatum*	K				[32]
*L. furfuraceum*	K				[32,35]
*L. punctulatum*	-				[32]
*L. rivulare*	T				[33]
*L. vesiculosum*	K, T				[32]

^1^ New observation for the country. ^2^ Reported as *Leptogium hibernicum* in [32,33].

## Data Availability

The data presented in this study are openly available in NCBI GenBank and the accession numbers are available in Table S1.

## Supplementary Materials

The following are available online at https://www.mdpi.com/2076-2607/9/2/314/s1, Figure S1: Length variation of the nuITS region in the different *Leptogium* clades, Figure S2: The complete phylogenetic tree inferred from the mtSSU and 5.8S regions, Table S1: Specimen information.
