# Supplementary material for: Diversity of Leptogium (Collemataceae, Ascomycota) in East African Montane Ecosystems
Source: Microorganisms. 2021 Feb 3;9(2):314. doi: 10.3390/microorganisms9020314 (PMC7913733; doi:10.3390/microorganisms9020314)
Supplement: Supplementary file 1 [file microorganisms-09-00314-s001.zip › Supplementary/FigureS1.pdf]

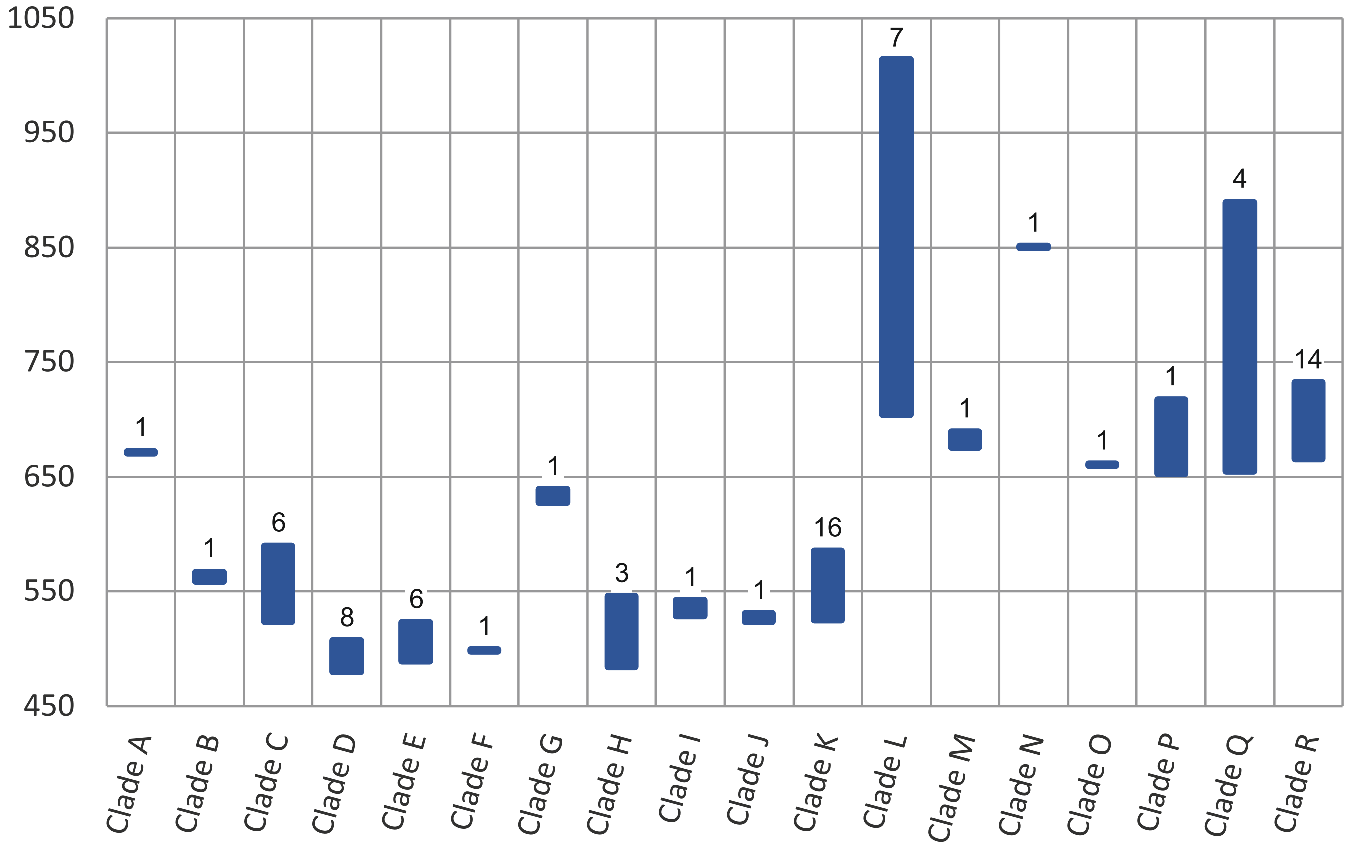

Figure S1. Length variation of the nuITS region in the different *Leptogium* clades (Figure S2) among the studied East African specimens. The length is shown on y-axis as base pairs, each bar ranging from the observed minimum to maximun length of the sequences in each clade. If full length nuITS regions could not be obtained, the lengths were estimated and/or based on partial sequences, in which case the here shown minimum and maximum lengths may be underestimating the true length of the nuITS regions (mainly in Clades L, N, and Q). The number of species/OTUs in each clade is shown above the bar.
