# Supplementary material for: Diversity of Leptogium (Collemataceae, Ascomycota) in East African Montane Ecosystems
Source: Microorganisms. 2021 Feb 3;9(2):314. doi: 10.3390/microorganisms9020314 (PMC7913733; doi:10.3390/microorganisms9020314)
Supplement: Supplementary file 1 [file microorganisms-09-00314-s001.zip › Supplementary/TableS1.pdf]

Kaasalainen *et al.* 2021: Diversity of *Leptogium* (Collemataceae, Ascomycota) in East African montane ecosystems. Supplementary Material.

Table S1. Collection information and GenBank Accession numbers for the mtSSU and nuITS sequences of the *Leptogium* specimens. The Clade refers to the clades shown in Figure S2.

Clade			Collection number	Collection locality	Forest/plot	NCBI Accession No. mtSSU	nuITS
Clade A	*Leptogium*	*burnetiae*	UK170858a	Tanzania, Mt. Kilimanjaro	FPo4	MW335165	MW340225
Clade B	*Leptogium*	sp.	JR10K131	Kenya, Mt. Kasigau	Kasigau N13	MW335166	MW340226
Clade B	*Leptogium*	sp.	JR10K571	Kenya, Mt. Kasigau	Kasigau 57W	MW335167	MW340227
Clade C	*Leptogium*	sp.	JR10K332	Kenya, Mt. Kasigau	Kasigau S1	MW335168	MW340228
Clade C	*Leptogium*	sp.	UK170998f	Tanzania, Mt. Kilimanjaro	Home 5	MW335169	MW340229
Clade C	*Leptogium*	sp.	UK171150g	Tanzania, Mt. Kilimanjaro	FED4	MW335170	-
Clade C	*Leptogium*	sp.	UK171397a	Tanzania, Mt. Kilimanjaro	FEr4	MW335171	MW340230
Clade C	*Leptogium*	OTU C1	JR10K091b	Kenya, Mt. Kasigau	Kasigau N9	MW335172	MW340231
Clade C	*Leptogium*	OTU C1	UK160487d	Tanzania, Mt. Kilimanjaro	home 4	MW335173	-
Clade C	*Leptogium*	OTU C2	JR10248B	Kenya, Taita Hills	Mwachora	MW335174	-
Clade C	*Leptogium*	OTU C2	JR10248C	Kenya, Taita Hills	Mwachora	MW335175	-
Clade C	*Leptogium*	OTU C2	JR10K171	Kenya, Mt. Kasigau	Kasigau E2	MW335176	MW340232
Clade C	*Leptogium*	OTU C2	JR10K232	Kenya, Mt. Kasigau	Kasigau E8	MW335177	MW340233
Clade C	*Leptogium*	OTU C2	UK170967	Tanzania, Mt. Kilimanjaro	Coffee 5	MW335178	MW340234
Clade D	*Leptogium*	*ethiopicum*	UK170821b	Tanzania, Mt. Kilimanjaro	FPD4	MW335179	MW340235
Clade D	*Leptogium*	*ethiopicum*	UK170845p	Tanzania, Mt. Kilimanjaro	FPD3	MW335180	MW340236
Clade D	*Leptogium*	*ethiopicum*	UK171473e	Tanzania, Mt. Kilimanjaro	FPD1	MW335181	MW340237
Clade D	*Leptogium*	*ethiopicum*	UK171486m	Tanzania, Mt. Kilimanjaro	FOc4	MW335182	-
Clade D	*Leptogium*	*ethiopicum*	UK171510f	Tanzania, Mt. Kilimanjaro	FOD2	MW335183	MW340238
Clade D	*Leptogium*	*ethiopicum*	UK171519j	Tanzania, Mt. Kilimanjaro	FOc5	MW335184	MW340239
Clade D	*Leptogium*	*ethiopicum*	UK171577o	Tanzania, Mt. Kilimanjaro	FPD2	MW335185	MW340240
Clade D	*Leptogium*	*ethiopicum*	UK171584f	Tanzania, Mt. Kilimanjaro	FPD2	MW335186	MW340241
Clade D	*Leptogium*	*ethiopicum*	UK171586z	Tanzania, Mt. Kilimanjaro	FPo3	MW335187	MW340242
Clade D	*Leptogium*	*juressianum*	JR10072B	Kenya, Taita Hills	Vuria	MW335188	JX503813
Clade D	*Leptogium*	*juressianum*	JR11030C	Kenya, Taita Hills	Vuria	-	JX503812
Clade D	*Leptogium*	*juressianum*	JR11031A	Kenya, Taita Hills	Vuria	MW335189	JX503814
Clade D	*Leptogium*	*juressianum*	JR11050C	Kenya, Taita Hills	Vuria	MW335190	JX503815
Clade D	*Leptogium*	*juressianum*	JR11067	Kenya, Taita Hills	Fururu	MW335191	JX503811
Clade D	*Leptogium*	*juressianum*	UK110535e	Tanzania, Mt. Kilimanjaro	Vuria	MW335192	-
Clade D	*Leptogium*	*juressianum*	UK170912d	Tanzania, Mt. Kilimanjaro	FOD5	MW335193	MW340243
Clade D	*Leptogium*	*juressianum*	UK170953a	Tanzania, Mt. Kilimanjaro	Flm1	MW335194	MW340244
Clade D	*Leptogium*	*juressianum*	UK171182h	Tanzania, Mt. Kilimanjaro	FOD3	MW335195	MW340245
Clade D	*Leptogium*	*krogiae*	JR_A45_R1	Kenya, Taita Hills	Ngangao	-	JX503786
Clade D	*Leptogium*	*krogiae*	JR10074A	Kenya, Taita Hills	Vuria	-	JX503796
Clade D	*Leptogium*	*krogiae*	JR10088B	Kenya, Taita Hills	Vuria	-	JX503795
Clade D	*Leptogium*	*krogiae*	JR10116A	Kenya, Taita Hills	Vuria	-	JX503793
Clade D	*Leptogium*	*krogiae*	JR10148	Kenya, Taita Hills	Ngangao	-	JX503784
Clade D	*Leptogium*	*krogiae*	JR10158B	Kenya, Taita Hills	Yale	-	JX503787
Clade D	*Leptogium*	*krogiae*	JR10182C	Kenya, Taita Hills	Yale	-	JX503797
Clade D	*Leptogium*	*krogiae*	JR10192A	Kenya, Taita Hills	Yale	-	JX503788
Clade D	*Leptogium*	*krogiae*	JR10199A	Kenya, Taita Hills	Yale	-	JX503798
Clade D	*Leptogium*	*krogiae*	JR10201A	Kenya, Taita Hills	Yale	-	JX503799
Clade D	*Leptogium*	*krogiae*	JR10204	Kenya, Taita Hills	Yale	-	JX503800
Clade D	*Leptogium*	*krogiae*	JR10210A	Kenya, Taita Hills	Yale	-	JX503801
Clade D	*Leptogium*	*krogiae*	JR10222A	Kenya, Taita Hills	Mwachora	-	JX503785
Clade D	*Leptogium*	*krogiae*	JR10226A	Kenya, Taita Hills	Mwachora	-	JX503791
Clade D	*Leptogium*	*krogiae*	JR10226B	Kenya, Taita Hills	Mwachora	-	JX503792
Clade D	*Leptogium*	*krogiae*	JR10242	Kenya, Taita Hills	Mwachora	-	JX503790
Clade D	*Leptogium*	*krogiae*	JR10252A	Kenya, Taita Hills	Mwachora	-	JX503794
Clade D	*Leptogium*	*krogiae*	JR10259A	Kenya, Taita Hills	Mwachora	-	JX503789
Clade D	*Leptogium*	*krogiae*	JR11012B	Kenya, Taita Hills	Vuria	MW335196	JX503802
Clade D	*Leptogium*	*krogiae*	JR11039A	Kenya, Taita Hills	Vuria	-	JX503803
Clade D	*Leptogium*	*krogiae*	JR11054A	Kenya, Taita Hills	Vuria	-	JX503804
Clade D	*Leptogium*	*krogiae*	UK110507c	Kenya, Taita Hills	Ngangao	MW335197	-

Clade			Collection number	Collection locality	Forest/plot	NCBI Accession No. mtSSU	nuITS
Clade D	*Leptogium*	*krogiae*	UK170804f	Tanzania, Mt. Kilimanjaro	FOc1	MW335198	MW340246
Clade D	*Leptogium*	*krogiae*	UK170882c	Tanzania, Mt. Kilimanjaro	FPo5	MW335199	MW340247
Clade D	*Leptogium*	*krogiae*	UK170897aq	Tanzania, Mt. Kilimanjaro	FOD4	MW335200	MW340248
Clade D	*Leptogium*	*krogiae*	UK170910a	Tanzania, Mt. Kilimanjaro	FOD5	MW335201	MW340249
Clade D	*Leptogium*	*krogiae*	UK170933a	Tanzania, Mt. Kilimanjaro	Flm1	MW335202	MW340250
Clade D	*Leptogium*	*krogiae*	UK171182g	Tanzania, Mt. Kilimanjaro	FOD3	MW335203	-
Clade D	*Leptogium*	*krogiae*	UK171480b	Tanzania, Mt. Kilimanjaro	FOc4	MW335204	MW340251
Clade D	*Leptogium*	*krogiae*	UK171486p	Tanzania, Mt. Kilimanjaro	FOc4	MW335205	MW340252
Clade D	*Leptogium*	*krogiae*	UK171489a	Tanzania, Mt. Kilimanjaro	FOc3	MW335206	MW340253
Clade D	*Leptogium*	*krogiae*	UK171497h	Tanzania, Mt. Kilimanjaro	FOD1	MW335207	MW340254
Clade D	*Leptogium*	*krogiae*	UK171505b	Tanzania, Mt. Kilimanjaro	FOD2	MW335208	MW340255
Clade D	*Leptogium*	*krogiae*	UK171508g	Tanzania, Mt. Kilimanjaro	FOD2	MW335209	MW340256
Clade D	*Leptogium*	*krogiae*	UK171519m	Tanzania, Mt. Kilimanjaro	FOc5	MW335210	MW340257
Clade D	*Leptogium*	*krogiae*	UK171523d	Tanzania, Mt. Kilimanjaro	FPo2	MW335211	MW340258
Clade D	*Leptogium*	*krogiae*	UK171577p	Tanzania, Mt. Kilimanjaro	FPD2	MW335212	MW340259
Clade D	*Leptogium*	*krogiae*	UK171586r	Tanzania, Mt. Kilimanjaro	FPo3	MW335213	MW340260
Clade D	*Leptogium*	*resupinans*	UK171433b	Tanzania, Mt. Kilimanjaro	FEr3	MW335214	MW340261
Clade D	*Leptogium*	*resupinans*	UK171438e	Tanzania, Mt. Kilimanjaro	FEr2	MW335215	MW340262
Clade D	*Leptogium*	*resupinans*	UK171458e	Tanzania, Mt. Kilimanjaro	FEr4	MW335216	MW340263
Clade D	*Leptogium*	sp.	UK171504q	Tanzania, Mt. Kilimanjaro	FOD2	MW335217	MW340264
Clade D	*Leptogium*	OTU D1	UK170774f	Tanzania, Mt. Kilimanjaro	FPo1	MW335218	MW340265
Clade D	*Leptogium*	OTU D1	UK171458f	Tanzania, Mt. Kilimanjaro	FEr4	MW335219	MW340266
Clade D	*Leptogium*	OTU D1	UK171468v	Tanzania, Mt. Kilimanjaro	FPD1	MW335220	MW340267
Clade D	*Leptogium*	OTU D1	UK171577l	Tanzania, Mt. Kilimanjaro	FPD2	MW335221	MW340268
Clade D	*Leptogium*	OTU D1	UK171586t	Tanzania, Mt. Kilimanjaro	FPo3	MW335222	MW340269
Clade D	*Leptogium*	OTU D2	JR010030F	Kenya, Taita Hills	Shomoto Hill	MW335223	-
Clade D	*Leptogium*	OTU D2	JR10072A	Kenya, Taita Hills	Vuria	MW335224	JX503807
Clade D	*Leptogium*	OTU D2	JR10104	Kenya, Taita Hills	Vuria	MW335225	JX503808
Clade D	*Leptogium*	OTU D2	JR11030B	Kenya, Taita Hills	Vuria	-	JX503809
Clade D	*Leptogium*	OTU D2	JR11034B	Kenya, Taita Hills	Vuria	-	JX503810
Clade D	*Leptogium*	OTU D2	UK170778d	Tanzania, Mt. Kilimanjaro	FPo1	MW335226	MW340270
Clade D	*Leptogium*	OTU D2	UK170781a	Tanzania, Mt. Kilimanjaro	FPo1	MW335227	MW340271
Clade D	*Leptogium*	OTU D2	UK170789a	Tanzania, Mt. Kilimanjaro	FOc2	MW335228	MW340272
Clade D	*Leptogium*	OTU D2	UK170806h	Tanzania, Mt. Kilimanjaro	FPD4	MW335229	MW340273
Clade D	*Leptogium*	OTU D2	UK170915c	Tanzania, Mt. Kilimanjaro	FOD5	MW335230	MW340274
Clade D	*Leptogium*	OTU D2	UK170922a	Tanzania, Mt. Kilimanjaro	Flm6	MW335231	MW340275
Clade D	*Leptogium*	OTU D2	UK170950j	Tanzania, Mt. Kilimanjaro	Flm1	MW335232	MW340276
Clade D	*Leptogium*	OTU D2	UK171181c	Tanzania, Mt. Kilimanjaro	FOD3	MW335233	MW340277
Clade D	*Leptogium*	OTU D2	UK171267b	Tanzania, Mt. Kilimanjaro	Hel1	MW335234	MW340278
Clade D	*Leptogium*	OTU D2	UK171340o	Tanzania, Mt. Kilimanjaro	FED1	MW335235	MW340279
Clade D	*Leptogium*	OTU D2	UK171472a	Tanzania, Mt. Kilimanjaro	FPD1	MW335236	MW340280
Clade D	*Leptogium*	OTU D2	UK171495c	Tanzania, Mt. Kilimanjaro	FOc3	MW335237	MW340281
Clade D	*Leptogium*	OTU D2	UK171510q	Tanzania, Mt. Kilimanjaro	FOD2	MW335238	MW340282
Clade D	*Leptogium*	OTU D2	UK171529e	Tanzania, Mt. Kilimanjaro	FPo2	MW335239	MW340283
Clade D	*Leptogium*	OTU D2	UK171586af	Tanzania, Mt. Kilimanjaro	FPo3	MW335240	MW340284
Clade D	*Leptogium*	OTU D3	JR10097Ab	Kenya, Taita Hills	Vuria	MW335241	-
Clade D	*Leptogium*	OTU D3	JR10252B	Kenya, Taita Hills	Mwachora	MW335242	JX503806
Clade D	*Leptogium*	OTU D3	UK170792f	Tanzania, Mt. Kilimanjaro	FOc2	MW335243	MW340285
Clade D	*Leptogium*	OTU D3	UK170794j	Tanzania, Mt. Kilimanjaro	FOc2	MW335244	MW340286
Clade D	*Leptogium*	OTU D3	UK170797d	Tanzania, Mt. Kilimanjaro	FOc1	MW335245	MW340287
Clade D	*Leptogium*	OTU D3	UK170900f	Tanzania, Mt. Kilimanjaro	FOD4	MW335246	MW340288
Clade D	*Leptogium*	OTU D3	UK170929a	Tanzania, Mt. Kilimanjaro	Flm6	MW335247	MW340289
Clade D	*Leptogium*	OTU D3	UK170935n	Tanzania, Mt. Kilimanjaro	Flm1	MW335248	MW340290
Clade D	*Leptogium*	OTU D3	UK171182e	Tanzania, Mt. Kilimanjaro	FOD3	MW335249	MW340291
Clade D	*Leptogium*	OTU D3	UK171185i	Tanzania, Mt. Kilimanjaro	FOD3	MW335250	MW340292
Clade D	*Leptogium*	OTU D3	UK171482b	Tanzania, Mt. Kilimanjaro	FOc4	MW335251	MW340293
Clade D	*Leptogium*	OTU D3	UK171494d	Tanzania, Mt. Kilimanjaro	FOc3	MW335252	MW340294
Clade D	*Leptogium*	OTU D3	UK171495b	Tanzania, Mt. Kilimanjaro	FOc3	MW335253	MW340295
Clade D	*Leptogium*	OTU D3	UK171505f	Tanzania, Mt. Kilimanjaro	FOD2	MW335254	MW340296
Clade D	*Leptogium*	OTU D3	UK171514f	Tanzania, Mt. Kilimanjaro	FOc5	MW335255	MW340297

Clade			Collection number	Collection locality	Forest/plot	NCBI Accession No. mtSSU	nuITS
Clade E	*Leptogium*	*caespitosum*	JR10014A	Kenya, Taita Hills	Taita Research Station	MW335256	JX503769
Clade E	*Leptogium*	*caespitosum*	JR10016	Kenya, Taita Hills	Taita Research Station	MW335257	JX503768
Clade E	*Leptogium*	*caespitosum*	JR10017b	Kenya, Taita Hills	Taita Research Station	MW335258	-
Clade E	*Leptogium*	*caespitosum*	JR10K011	Kenya, Mt. Kasigau	Kasigau 1N	MW335259	-
Clade E	*Leptogium*	*caespitosum*	JR10K061B	Kenya, Mt. Kasigau	Kasigau 6N	MW335260	MW340298
Clade E	*Leptogium*	*caespitosum*	JR10K071b	Kenya, Mt. Kasigau	Kasigau 7N	MW335261	MW340299
Clade E	*Leptogium*	*caespitosum*	JR10K231	Kenya, Mt. Kasigau	Kasigau 23E	MW335262	MW340300
Clade E	*Leptogium*	*caespitosum*	JR10K401A	Kenya, Mt. Kasigau	Kasigau 40S	MW335263	MW340301
Clade E	*Leptogium*	*caespitosum*	JR10K401B	Kenya, Mt. Kasigau	Kasigau 40S	MW335264	MW340302
Clade E	*Leptogium*	*caespitosum*	JR10K402c	Kenya, Mt. Kasigau	Kasigau 40S	-	MW340303
Clade E	*Leptogium*	*caespitosum*	JR10K412	Kenya, Mt. Kasigau	Kasigau 41S	MW335265	-
Clade E	*Leptogium*	*marginellum*	JR10K251A	Kenya, Mt. Kasigau	Kasigau	MW335266	MW340304
Clade E	*Leptogium*	sp.	JR11094A	Kenya, Taita Hills	Chawia	-	JX503818
Clade E	*Leptogium*	OTU E1	JR10013B	Kenya, Taita Hills	Taita Research Station	MW335267	JX503820
Clade E	*Leptogium*	OTU E1	JR10061	Kenya, Taita Hills	Shomoto Hill	MW335268	JX503819
Clade E	*Leptogium*	OTU E1	JR10K042	Kenya, Mt. Kasigau	Kasigau N4	MW335269	MW340305
Clade E	*Leptogium*	OTU E1	JR10K192	Kenya, Mt. Kasigau	Kasigau E4	MW335270	MW340306
Clade E	*Leptogium*	OTU E1	UK160414b	Tanzania, Mt. Kilimanjaro	Savanna 1	MW335271	MW340307
Clade E	*Leptogium*	OTU E1	UK160424a	Tanzania, Mt. Kilimanjaro	Savanna 1	MW335272	-
Clade E	*Leptogium*	OTU E1	UK160440a	Tanzania, Mt. Kilimanjaro	Savanna 2	MW335273	MW340308
Clade E	*Leptogium*	OTU E1	UK170999a	Tanzania, Mt. Kilimanjaro	Home 5	MW335274	MW340309
Clade E	*Leptogium*	OTU E1	UK171007a	Tanzania, Mt. Kilimanjaro	Grass 5	MW335275	MW340310
Clade E	*Leptogium*	OTU E2	JR10020	Kenya, Taita Hills	Taita Research Station	MW335276	JX503817
Clade E	*Leptogium*	OTU E2	JR10041Ab	Kenya, Taita Hills	Shomoto Hill	MW335277	-
Clade E	*Leptogium*	OTU E2	JR10K012	Kenya, Mt. Kasigau	Kasigau N1	-	JX503816
Clade E	*Leptogium*	OTU E2	JR10K234	Kenya, Mt. Kasigau	Kasigau E8	MW335278	MW340311
Clade E	*Leptogium*	OTU E2	UK170994a	Tanzania, Mt. Kilimanjaro	Home 5	MW335279	MW340312
Clade E	*Leptogium*	OTU E3	JR_X7B	Kenya, Taita Hills	Chawia	-	JX503760
Clade E	*Leptogium*	OTU E3	JR10016E	Kenya, Taita Hills	Taita Research Station	MW335280	-
Clade E	*Leptogium*	OTU E3	JR10059	Kenya, Taita Hills	Shomoto Hill	-	JX503765
Clade E	*Leptogium*	OTU E3	JR10081B	Kenya, Taita Hills	Vuria	MW335281	-
Clade E	*Leptogium*	OTU E3	JR10100	Kenya, Taita Hills	Vuria	-	JX503771
Clade E	*Leptogium*	OTU E3	JR10120A	Kenya, Taita Hills	Vuria	MW335282	JX503772
Clade E	*Leptogium*	OTU E3	JR10123A	Kenya, Taita Hills	Vuria	MW335283	JX503773
Clade E	*Leptogium*	OTU E3	JR10123B	Kenya, Taita Hills	Vuria	MW335284	-
Clade E	*Leptogium*	OTU E3	JR10124	Kenya, Taita Hills	Vuria	MW335285	JX503774
Clade E	*Leptogium*	OTU E3	JR10126	Kenya, Taita Hills	Vuria	-	JX503770
Clade E	*Leptogium*	OTU E3	JR10156A	Kenya, Taita Hills	Yale	-	JX503761
Clade E	*Leptogium*	OTU E3	JR10156B	Kenya, Taita Hills	Yale	MW335286	JX503762
Clade E	*Leptogium*	OTU E3	JR10158A	Kenya, Taita Hills	Yale	MW335287	JX503766
Clade E	*Leptogium*	OTU E3	JR10163A	Kenya, Taita Hills	Yale	MW335288	-
Clade E	*Leptogium*	OTU E3	JR10169A	Kenya, Taita Hills	Yale	MW335289	JX503763
Clade E	*Leptogium*	OTU E3	JR10170B	Kenya, Taita Hills	Yale	MW335290	JX503758
Clade E	*Leptogium*	OTU E3	JR10170C	Kenya, Taita Hills	Yale	MW335291	JX503767
Clade E	*Leptogium*	OTU E3	JR10182B	Kenya, Taita Hills	Yale	MW335292	-
Clade E	*Leptogium*	OTU E3	JR10260B	Kenya, Taita Hills	Mwachora	-	JX503764
Clade E	*Leptogium*	OTU E3	JR10283A	Kenya, Taita Hills	Macha	MW335293	JX503759
Clade E	*Leptogium*	OTU E3	UK110514c	Kenya, Taita Hills	Vuria	MW335294	-
Clade E	*Leptogium*	OTU E3	UK170778e	Tanzania, Mt. Kilimanjaro	FPo1	MW335295	MW340313
Clade E	*Leptogium*	OTU E3	UK170804e	Tanzania, Mt. Kilimanjaro	FOc1	-	MW340314
Clade E	*Leptogium*	OTU E3	UK170845r	Tanzania, Mt. Kilimanjaro	FPD3	MW335296	MW340315
Clade E	*Leptogium*	OTU E3	UK170944a	Tanzania, Mt. Kilimanjaro	Flm1	MW335297	MW340316
Clade E	*Leptogium*	OTU E3	UK171185m	Tanzania, Mt. Kilimanjaro	FOD3	MW335298	MW340317
Clade E	*Leptogium*	OTU E3	UK171497a	Tanzania, Mt. Kilimanjaro	FOD1	MW335299	MW340318
Clade E	*Leptogium*	OTU E3	UK171505e	Tanzania, Mt. Kilimanjaro	FOD2	MW335300	MW340319
Clade E	*Leptogium*	OTU E3	UK171510p	Tanzania, Mt. Kilimanjaro	FOD2	MW335301	MW340320
Clade E	*Leptogium*	OTU E3	UK171577r	Tanzania, Mt. Kilimanjaro	FPD2	MW335302	MW340321
Clade E	*Leptogium*	OTU E3	UK171582h	Tanzania, Mt. Kilimanjaro	FPD2	MW335303	MW340322
Clade E	*Leptogium*	OTU E3	UK171591a	Tanzania, Mt. Kilimanjaro	FPo3	MW335304	MW340323
Clade E	*Leptogium*	OTU E3	UK171591e	Tanzania, Mt. Kilimanjaro	FPo3	MW335305	MW340324

Clade			Collection number	Collection locality	Forest/plot	NCBI Accession No. mtSSU	nuITS
Clade F	*Leptogium*	OTU F1	JR10120C	Kenya, Taita Hills	Vuria	MW335306	JX503839
Clade F	*Leptogium*	OTU F1	JR10120D	Kenya, Taita Hills	Vuria	MW335307	-
Clade F	*Leptogium*	OTU F1	JR10170D	Kenya, Taita Hills	Yale	-	JX503840
Clade F	*Leptogium*	OTU F1	UK170855b	Tanzania, Mt. Kilimanjaro	FPo4	MW335308	MW340325
Clade G	*Leptogium*	OTU G1	UK170845s	Tanzania, Mt. Kilimanjaro	FPD3	MW335309	MW340326
Clade G	*Leptogium*	OTU G1	UK170858h	Tanzania, Mt. Kilimanjaro	FPo4	MW335310	MW340327
Clade G	*Leptogium*	OTU G1	UK171185o	Tanzania, Mt. Kilimanjaro	FOD3	MW335311	MW340328
Clade G	*Leptogium*	OTU G1	UK171505g	Tanzania, Mt. Kilimanjaro	FOD2	MW335312	MW340329
Clade H	*Leptogium*	OTU H1	JR10017a	Kenya, Taita Hills	Taita Research Station	MW335313	JX503805
Clade H	*Leptogium*	OTU H1	UK160418b	Tanzania, Mt. Kilimanjaro	savanna 1	MW335314	-
Clade H	*Leptogium*	OTU H1	UK160418d	Tanzania, Mt. Kilimanjaro	savanna 1	MW335315	-
Clade H	*Leptogium*	OTU H2	UK170792g	Tanzania, Mt. Kilimanjaro	FOc2	MW335316	MW340330
Clade H	*Leptogium*	OTU H2	UK170821a	Tanzania, Mt. Kilimanjaro	FPD4	MW335317	MW340331
Clade H	*Leptogium*	OTU H2	UK170880n	Tanzania, Mt. Kilimanjaro	FPo5	MW335318	MW340332
Clade H	*Leptogium*	OTU H2	UK170911a	Tanzania, Mt. Kilimanjaro	FOD5	MW335319	MW340333
Clade H	*Leptogium*	OTU H2	UK171340j	Tanzania, Mt. Kilimanjaro	FED1	MW335320	MW340334
Clade H	*Leptogium*	OTU H2	UK171439e	Tanzania, Mt. Kilimanjaro	FEr2	MW335321	MW340335
Clade H	*Leptogium*	OTU H2	UK171478e	Tanzania, Mt. Kilimanjaro	FOc4	MW335322	MW340336
Clade H	*Leptogium*	OTU H2	UK171494b	Tanzania, Mt. Kilimanjaro	FOc3	MW335323	MW340337
Clade H	*Leptogium*	OTU H2	UK171516y	Tanzania, Mt. Kilimanjaro	FOc5	MW335324	MW340338
Clade H	*Leptogium*	OTU H2	UK171525k	Tanzania, Mt. Kilimanjaro	FPo2	MW335325	MW340339
Clade H	*Leptogium*	OTU H2	UK171584c	Tanzania, Mt. Kilimanjaro	FPD2	MW335326	MW340340
Clade H	*Leptogium*	OTU H3	JR10077	Kenya, Taita Hills	Vuria	MW335327	JX503836
Clade H	*Leptogium*	OTU H3	JR10081A	Kenya, Taita Hills	Vuria	MW335328	JX503838
Clade H	*Leptogium*	OTU H3	JR10119Bb	Kenya, Taita Hills	Vuria	MW335329	-
Clade H	*Leptogium*	OTU H3	JR10163B	Kenya, Taita Hills	Yale	MW335330	JX503837
Clade H	*Leptogium*	OTU H3	UK170927h	Tanzania, Mt. Kilimanjaro	Flm6	MW335331	MW340341
Clade H	*Leptogium*	OTU H3	UK170998c	Tanzania, Mt. Kilimanjaro	Home 5	MW335332	MW340342
Clade I	*Leptogium*	*austroamericanum*	JR_W8b	Kenya, Mt. Kasigau	Kasigau	MW335333	-
Clade I	*Leptogium*	*austroamericanum*	JR290B	Kenya, Mt. Kasigau	Kasigau N-slope	MW335353	-
Clade I	*Leptogium*	*austroamericanum*	JR10K033	Kenya, Mt. Kasigau	Kasigau N3	MW335334	MW340343
Clade I	*Leptogium*	*austroamericanum*	JR10K041	Kenya, Mt. Kasigau	Kasigau N4	MW335335	MW340344
Clade I	*Leptogium*	*austroamericanum*	JR10K084Aa	Kenya, Mt. Kasigau	Kasigau N8	MW335336	MW340345
Clade I	*Leptogium*	*austroamericanum*	JR10K092	Kenya, Mt. Kasigau	Kasigau N9	MW335337	MW340346
Clade I	*Leptogium*	*austroamericanum*	JR10K111c	Kenya, Mt. Kasigau	Kasigau N11	MW335338	MW340347
Clade I	*Leptogium*	*austroamericanum*	JR10K201	Kenya, Mt. Kasigau	Kasigau E5	MW335339	MW340348
Clade I	*Leptogium*	*austroamericanum*	JR10K202	Kenya, Mt. Kasigau	Kasigau E5	MW335340	MW340349
Clade I	*Leptogium*	*austroamericanum*	JR10K211Ba	Kenya, Mt. Kasigau	Kasigau E6	MW335341	MW340350
Clade I	*Leptogium*	*austroamericanum*	JR10K251C	Kenya, Mt. Kasigau	Kasigau 25E	MW335342	-
Clade I	*Leptogium*	*austroamericanum*	JR10K341a	Kenya, Mt. Kasigau	Kasigau S2	MW335343	MW340351
Clade I	*Leptogium*	*austroamericanum*	JR10K351	Kenya, Mt. Kasigau	Kasigau S3	MW335344	MW340352
Clade I	*Leptogium*	*austroamericanum*	JR10K361	Kenya, Mt. Kasigau	Kasigau S4	MW335345	MW340353
Clade I	*Leptogium*	*austroamericanum*	JR10K362	Kenya, Mt. Kasigau	Kasigau S4	MW335346	MW340354
Clade I	*Leptogium*	*austroamericanum*	JR10K381	Kenya, Mt. Kasigau	Kasigau S6	MW335347	MW340355
Clade I	*Leptogium*	*austroamericanum*	JR10K391	Kenya, Mt. Kasigau	Kasigau S7	MW335348	MW340356
Clade I	*Leptogium*	*austroamericanum*	JR10K422	Kenya, Mt. Kasigau	Kasigau S10	MW335349	MW340357
Clade I	*Leptogium*	*austroamericanum*	JR10K432	Kenya, Mt. Kasigau	Kasigau S11	MW335350	MW340358
Clade I	*Leptogium*	*austroamericanum*	JR10K461	Kenya, Mt. Kasigau	Kasigau W2	MW335351	MW340359
Clade I	*Leptogium*	*austroamericanum*	JR10K491A	Kenya, Mt. Kasigau	Kasigau W5	MW335352	MW340360
Clade J	*Leptogium*	OTU J1	JR_AT1	Kenya, Taita Hills	Ngangao	MW335354	JX503834
Clade J	*Leptogium*	OTU J1	JR_AT2A	Kenya, Taita Hills	Ngangao	MW335355	JX503835
Clade J	*Leptogium*	OTU J1	JR_D12_B1	Kenya, Taita Hills	Ngangao	-	JX503824
Clade J	*Leptogium*	OTU J1	JR_X1	Kenya, Taita Hills	Chawia	MW335356	JX503823
Clade J	*Leptogium*	OTU J1	JR10012A	Kenya, Taita Hills	Taita Research Station	MW335357	-
Clade J	*Leptogium*	OTU J1	JR10036	Kenya, Taita Hills	Shomoto Hill	MW335358	-
Clade J	*Leptogium*	OTU J1	JR10097A	Kenya, Taita Hills	Vuria	MW335359	-
Clade J	*Leptogium*	OTU J1	JR10097B	Kenya, Taita Hills	Vuria	MW335360	JX503822
Clade J	*Leptogium*	OTU J1	JR10128B	Kenya, Taita Hills	Vuria	MW335361	JX503826
Clade J	*Leptogium*	OTU J1	JR10146	Kenya, Taita Hills	Ngangao	MW335362	JX503832
Clade J	*Leptogium*	OTU J1	JR10186A	Kenya, Taita Hills	Yale	MW335363	JX503825

Clade			Collection number	Collection locality	Forest/plot	NCBI Accession No. mtSSU	nuITS
Clade J	*Leptogium*	OTU J1	JR10186C	Kenya, Taita Hills	Yale	-	JX503827
Clade J	*Leptogium*	OTU J1	JR10232B	Kenya, Taita Hills	Mwachora	MW335364	JX503830
Clade J	*Leptogium*	OTU J1	JR10K032	Kenya, Mt. Kasigau	Kasigau N3	MW335365	MW340361
Clade J	*Leptogium*	OTU J1	JR10K251B	Kenya, Mt. Kasigau	Kasigau E10	MW335366	MW340362
Clade J	*Leptogium*	OTU J1	JR10K258	Kenya, Mt. Kasigau	Kasigau 25-26 E	MW335367	-
Clade J	*Leptogium*	OTU J1	JR10K261a	Kenya, Mt. Kasigau	Kasigau E11	MW335368	MW340363
Clade J	*Leptogium*	OTU J1	JR10K261b	Kenya, Mt. Kasigau	Kasigau E11	MW335369	MW340364
Clade J	*Leptogium*	OTU J1	JR10K481	Kenya, Mt. Kasigau	Kasigau 48W	MW335370	MW340365
Clade J	*Leptogium*	OTU J1	JR10K491B	Kenya, Mt. Kasigau	Kasigau 49W	MW335371	MW340366
Clade J	*Leptogium*	OTU J1	JR10K491C	Kenya, Mt. Kasigau	Kasigau 49W	MW335372	MW340367
Clade J	*Leptogium*	OTU J1	JR10K492b	Kenya, Mt. Kasigau	Kasigau 49W	MW335373	MW340368
Clade J	*Leptogium*	OTU J1	JR10K551A	Kenya, Mt. Kasigau	Kasigau 55W	MW335374	MW340369
Clade J	*Leptogium*	OTU J1	JR10K551B	Kenya, Mt. Kasigau	Kasigau 55W	MW335375	MW340370
Clade J	*Leptogium*	OTU J1	JR10K581A	Kenya, Mt. Kasigau	Kasigau 58W	MW335376	MW340371
Clade J	*Leptogium*	OTU J1	JR10K581B	Kenya, Mt. Kasigau	Kasigau 58W	MW335377	MW340372
Clade J	*Leptogium*	OTU J1	JR11006A	Kenya, Taita Hills	Vuria	MW335378	JX503833
Clade J	*Leptogium*	OTU J1	JR11009A	Kenya, Taita Hills	Vuria	MW335379	JX503829
Clade J	*Leptogium*	OTU J1	JR11010Aa	Kenya, Taita Hills	Vuria	MW335380	JX503821
Clade J	*Leptogium*	OTU J1	UK110521	Kenya, Taita Hills	Vuria	MW335381	JX503828
Clade J	*Leptogium*	OTU J1	UK170804c	Tanzania, Mt. Kilimanjaro	FOc1	MW335382	MW340373
Clade J	*Leptogium*	OTU J1	UK170917a	Tanzania, Mt. Kilimanjaro	Flm6	MW335383	MW340374
Clade J	*Leptogium*	OTU J1	UK170920b	Tanzania, Mt. Kilimanjaro	Flm6	MW335384	MW340375
Clade J	*Leptogium*	OTU J1	UK170929e	Tanzania, Mt. Kilimanjaro	Flm6	MW335385	MW340376
Clade J	*Leptogium*	OTU J1	UK170950f	Tanzania, Mt. Kilimanjaro	Flm1	MW335386	MW340377
Clade J	*Leptogium*	OTU J1	UK170951e	Tanzania, Mt. Kilimanjaro	Flm1	MW335387	MW340378
Clade J	*Leptogium*	OTU J1	UK171201a	Tanzania, Mt. Kilimanjaro	Flm3	MW335388	MW340379
Clade J	*Leptogium*	OTU J1	UK171204b	Tanzania, Mt. Kilimanjaro	Flm3	MW335389	MW340380
Clade J	*Leptogium*	OTU J1	UK171204c	Tanzania, Mt. Kilimanjaro	Flm3	MW335390	MW340381
Clade J	*Leptogium*	OTU J1	UK171483j	Tanzania, Mt. Kilimanjaro	FOc4	MW335391	MW340382
Clade J	*Leptogium*	OTU J1	UK171498c	Tanzania, Mt. Kilimanjaro	FOD1	MW335392	MW340383
Clade J	*Leptogium*	OTU J1	UK171502a	Tanzania, Mt. Kilimanjaro	FOD1	MW335393	MW340384
Clade J	*Leptogium*	OTU J1	UK171590p	Tanzania, Mt. Kilimanjaro	FPo3	MW335394	MW340385
Clade J	*Leptogium*	OTU J1	UK171593	Tanzania, Mt. Kilimanjaro	Flm2	MW335395	MW340386
Clade J	*Leptogium*	OTU J1	UK171596g	Tanzania, Mt. Kilimanjaro	Flm2	MW335396	MW340387
Clade K	*Leptogium*	*javanicum*	JR10110A	Kenya, Taita Hills	Vuria	-	JX503864
Clade K	*Leptogium*	*javanicum*	JR10113A	Kenya, Taita Hills	Vuria	-	JX503866
Clade K	*Leptogium*	*javanicum*	JR10113B	Kenya, Taita Hills	Vuria	MW335397	-
Clade K	*Leptogium*	*javanicum*	JR10113C	Kenya, Taita Hills	Vuria	MW335398	-
Clade K	*Leptogium*	*javanicum*	JR10115A	Kenya, Taita Hills	Vuria	-	JX503865
Clade K	*Leptogium*	*javanicum*	JR10119B	Kenya, Taita Hills	Vuria	MW335399	JX503867
Clade K	*Leptogium*	*javanicum*	JR10131A	Kenya, Taita Hills	Vuria	MW335400	JX503863
Clade K	*Leptogium*	*javanicum*	JR10131B	Kenya, Taita Hills	Vuria	-	JX503862
Clade K	*Leptogium*	sp.	JR10251C	Kenya, Taita Hills	Mwachora	MW335401	JX503846
Clade K	*Leptogium*	OTU K1	JR_W8a	Kenya, Mt. Kasigau	Kasigau	MW335402	JX503841
Clade K	*Leptogium*	OTU K1	JR10K423b	Kenya, Mt. Kasigau	Kasigau S10-11	MW335403	MW340388
Clade K	*Leptogium*	OTU K1	UK171189b	Tanzania, Mt. Kilimanjaro	Flm4	MW335404	MW340389
Clade K	*Leptogium*	OTU K1	UK171198a	Tanzania, Mt. Kilimanjaro	Flm4	MW335405	MW340390
Clade K	*Leptogium*	OTU K2	JR10K533	Kenya, Mt. Kasigau	Kasigau W9-10	MW335406	MW340391
Clade K	*Leptogium*	OTU K2	JR10K561	Kenya, Mt. Kasigau	Kasigau W12	MW335407	MW340392
Clade K	*Leptogium*	OTU K3	JR_Y15	Kenya, Taita Hills	Ngangao	-	JX503845
Clade K	*Leptogium*	OTU K3	JR10094	Kenya, Taita Hills	Vuria	MW335408	-
Clade K	*Leptogium*	OTU K3	JR10096	Kenya, Taita Hills	Vuria	MW335409	JX503844
Clade K	*Leptogium*	OTU K3	JR10098A	Kenya, Taita Hills	Vuria	MW335410	JX503842
Clade K	*Leptogium*	OTU K3	JR10098B	Kenya, Taita Hills	Vuria	-	JX503843
Clade K	*Leptogium*	OTU K3	JR10K511	Kenya, Mt. Kasigau	Kasigau W7	MW335411	MW340393
Clade K	*Leptogium*	OTU K3	UK110530b	Kenya, Taita Hills	Vuria	MW335412	-
Clade K	*Leptogium*	OTU K3	UK170897d	Tanzania, Mt. Kilimanjaro	FOD4	MW335413	MW340394
Clade K	*Leptogium*	OTU K3	UK170897e	Tanzania, Mt. Kilimanjaro	FOD4	MW335414	MW340395
Clade K	*Leptogium*	OTU K3	UK170900b	Tanzania, Mt. Kilimanjaro	FOD4	MW335415	MW340396
Clade K	*Leptogium*	OTU K3	UK170909c	Tanzania, Mt. Kilimanjaro	FOD5	MW335416	MW340397

Clade			Collection number	Collection locality	Forest/plot	NCBI Accession No. mtSSU	nuITS
Clade K	*Leptogium*	OTU K3	UK170912f	Tanzania, Mt. Kilimanjaro	FOD5	MW335417	MW340398
Clade K	*Leptogium*	OTU K3	UK170914a	Tanzania, Mt. Kilimanjaro	FOD5	MW335418	MW340399
Clade K	*Leptogium*	OTU K3	UK170920a	Tanzania, Mt. Kilimanjaro	Flm6	MW335419	MW340400
Clade K	*Leptogium*	OTU K3	UK170921a	Tanzania, Mt. Kilimanjaro	Flm6	MW335420	MW340401
Clade K	*Leptogium*	OTU K3	UK170925a	Tanzania, Mt. Kilimanjaro	Flm6	MW335421	MW340402
Clade K	*Leptogium*	OTU K3	UK170935r	Tanzania, Mt. Kilimanjaro	Flm1	MW335422	MW340403
Clade K	*Leptogium*	OTU K3	UK171483k	Tanzania, Mt. Kilimanjaro	FOc4	MW335423	MW340404
Clade K	*Leptogium*	OTU K3	UK171483m	Tanzania, Mt. Kilimanjaro	FOc4	MW335424	MW340405
Clade K	*Leptogium*	OTU K3	UK171497b	Tanzania, Mt. Kilimanjaro	FOD1	MW335425	MW340406
Clade K	*Leptogium*	OTU K3	UK171597b	Tanzania, Mt. Kilimanjaro	Flm2	MW335426	MW340407
Clade K	*Leptogium*	OTU K4	JR10158C	Kenya, Taita Hills	Yale	MW335427	JX503847
Clade K	*Leptogium*	OTU K4	JR10177C	Kenya, Taita Hills	Yale	MW335428	JX503848
Clade K	*Leptogium*	OTU K4	JR10259B	Kenya, Taita Hills	Mwachora	MW335429	JX503849
Clade K	*Leptogium*	OTU K4	JR10259C	Kenya, Taita Hills	Mwachora	MW335430	-
Clade K	*Leptogium*	OTU K4	UK110564	Kenya, Taita Hills	Chawia	MW335431	-
Clade K	*Leptogium*	OTU K5	JR11007B	Kenya, Taita Hills	Vuria	MW335432	-
Clade K	*Leptogium*	OTU K5	JR10030A	Kenya, Taita Hills	Shomoto Hill	MW335434	JX503853
Clade K	*Leptogium*	OTU K5	JR10033A	Kenya, Taita Hills	Shomoto Hill	MW335435	JX503854
Clade K	*Leptogium*	OTU K5	JR10239A	Kenya, Taita Hills	Mwachora	-	JX503852
Clade K	*Leptogium*	OTU K5	JR10251A	Kenya, Taita Hills	Mwachora	MW335436	JX503856
Clade K	*Leptogium*	OTU K5	JR10K111b	Kenya, Mt. Kasigau	Kasigau N11	MW335437	MW340408
Clade K	*Leptogium*	OTU K5	JR11026	Kenya, Taita Hills	Vuria	MW335438	JX503858
Clade K	*Leptogium*	OTU K5	JR11002A	Kenya, Taita Hills	Werugha	-	JX503857
Clade K	*Leptogium*	OTU K5	JR10016F	Kenya, Taita Hills	Vuria	MW335433	-
Clade K	*Leptogium*	OTU K5	JR11054D	Kenya, Taita Hills	Vuria	-	JX503855
Clade K	*Leptogium*	OTU K5	JR11060	Kenya, Taita Hills	Fururu	-	JX503851
Clade K	*Leptogium*	OTU K5	UK110513	Kenya, Taita Hills	Vuria	MW335439	JX503859
Clade K	*Leptogium*	OTU K6	JR_X7A	Kenya, Taita Hills	Chawia	MW335440	JX503850
Clade K	*Leptogium*	OTU K6	JR10K272	Kenya, Mt. Kasigau	Kasigau E12	MW335441	MW340409
Clade K	*Leptogium*	OTU K7	JR10110B	Kenya, Taita Hills	Vuria	MW335442	-
Clade K	*Leptogium*	OTU K7	JR10125	Kenya, Taita Hills	Vuria	MW335443	JX503861
Clade K	*Leptogium*	OTU K8	JR10145A	Kenya, Taita Hills	Ngangao	MW335444	JX503860
Clade K	*Leptogium*	OTU K8	JR10145C	Kenya, Taita Hills	Ngangao	MW335445	-
Clade K	*Leptogium*	OTU K8	UK170951d	Tanzania, Mt. Kilimanjaro	Flm1	MW335446	MW340410
Clade K	*Leptogium*	OTU K8	UK171596h	Tanzania, Mt. Kilimanjaro	Flm2	MW335447	MW340411
Clade K	*Leptogium*	OTU K9	JR10K031	Kenya, Mt. Kasigau	Kasigau N3	MW335448	MW340412
Clade K	*Leptogium*	OTU K9	JR10K181a	Kenya, Mt. Kasigau	Kasigau E3	MW335449	MW340413
Clade K	*Leptogium*	OTU K9	JR10K182	Kenya, Mt. Kasigau	Kasigau E3	MW335450	MW340414
Clade K	*Leptogium*	OTU K9	JR10K371	Kenya, Mt. Kasigau	Kasigau S5	MW335451	MW340415
Clade K	*Leptogium*	OTU K9	JR10K392B2	Kenya, Mt. Kasigau	Kasigau S7	MW335477	MW340416
Clade K	*Leptogium*	OTU K9	JR10K451b	Kenya, Mt. Kasigau	Kasigau W1	MW335453	MW340417
Clade K	*Leptogium*	OTU K9	JR10K452b	Kenya, Mt. Kasigau	Kasigau W1	MW335454	MW340418
Clade K	*Leptogium*	OTU K10	JR10021	Kenya, Taita Hills	Taita Research Station	MW335455	-
Clade K	*Leptogium*	OTU K10	JR10022C	Kenya, Taita Hills	Taita Research Station	-	JX503869
Clade K	*Leptogium*	OTU K10	JR10063B	Kenya, Taita Hills	Shomoto Hill	MW335456	-
Clade K	*Leptogium*	OTU K10	JR10063C	Kenya, Taita Hills	Shomoto Hill	MW335457	-
Clade K	*Leptogium*	OTU K10	JR10110C	Kenya, Taita Hills	Vuria	-	JX503871
Clade K	*Leptogium*	OTU K10	JR10128A	Kenya, Taita Hills	Vuria	-	JX503872
Clade K	*Leptogium*	OTU K10	JR10149B	Kenya, Taita Hills	Yale	MW335458	JX503881
Clade K	*Leptogium*	OTU K10	JR10153A	Kenya, Taita Hills	Yale	MW335459	JX503882
Clade K	*Leptogium*	OTU K10	JR10169C	Kenya, Taita Hills	Yale	MW335460	JX503868
Clade K	*Leptogium*	OTU K10	JR10174A	Kenya, Taita Hills	Yale	MW335461	-
Clade K	*Leptogium*	OTU K10	JR10174B	Kenya, Taita Hills	Yale	MW335462	-
Clade K	*Leptogium*	OTU K10	JR10174C	Kenya, Taita Hills	Yale	MW335463	-
Clade K	*Leptogium*	OTU K10	JR10178B	Kenya, Taita Hills	Yale	-	JX503873
Clade K	*Leptogium*	OTU K10	JR10185A	Kenya, Taita Hills	Yale	MW335464	JX503883
Clade K	*Leptogium*	OTU K10	JR10197	Kenya, Taita Hills	Yale	MW335465	JX503870
Clade K	*Leptogium*	OTU K10	JR10199B	Kenya, Taita Hills	Yale	MW335466	JX503877
Clade K	*Leptogium*	OTU K10	JR10221A	Kenya, Taita Hills	Mwachora	-	JX503874
Clade K	*Leptogium*	OTU K10	JR10221B	Kenya, Taita Hills	Mwachora	MW335467	JX503875

Clade			Collection number	Collection locality	Forest/plot	NCBI Accession No. mtSSU	nuITS
Clade K	*Leptogium*	OTU K10	JR10235A	Kenya, Taita Hills	Mwachora	MW335468	-
Clade K	*Leptogium*	OTU K10	JR10269A	Kenya, Taita Hills	Mwachora	-	JX503879
Clade K	*Leptogium*	OTU K10	JR10269B	Kenya, Taita Hills	Mwachora	-	JX503880
Clade K	*Leptogium*	OTU K10	JR10283C	Kenya, Taita Hills	Macha	MW335469	JX503878
Clade K	*Leptogium*	OTU K10	JR10K063	Kenya, Mt. Kasigau	Kasigau N6	MW335470	MW340419
Clade K	*Leptogium*	OTU K10	JR10K071a	Kenya, Mt. Kasigau	Kasigau N7	MW335471	MW340420
Clade K	*Leptogium*	OTU K10	JR10K121a	Kenya, Mt. Kasigau	Kasigau N12	MW335472	MW340421
Clade K	*Leptogium*	OTU K10	JR10K211A1	Kenya, Mt. Kasigau	Kasigau E6	MW335473	MW340422
Clade K	*Leptogium*	OTU K10	JR10K211Bb	Kenya, Mt. Kasigau	Kasigau E6	MW335474	MW340423
Clade K	*Leptogium*	OTU K10	JR10K242	Kenya, Mt. Kasigau	Kasigau E9	MW335475	MW340424
Clade K	*Leptogium*	OTU K10	JR10K245	Kenya, Mt. Kasigau	Kasigau E9-10	MW335476	MW340425
Clade K	*Leptogium*	OTU K10	JR10K392B	Kenya, Mt. Kasigau	Kasigau S7	MW335452	MW340426
Clade K	*Leptogium*	OTU K10	JR10K471	Kenya, Mt. Kasigau	Kasigau W3	MW335478	MW340427
Clade K	*Leptogium*	OTU K10	JR10K531B	Kenya, Mt. Kasigau	Kasigau W9	MW335479	MW340428
Clade K	*Leptogium*	OTU K10	JR11078A	Kenya, Taita Hills	Chawia	MW335480	JX503876
Clade K	*Leptogium*	OTU K11	JR10040B	Kenya, Taita Hills	Shomoto Hill	-	JX503884
Clade K	*Leptogium*	OTU K11	JR10040C	Kenya, Taita Hills	Shomoto Hill	MW335481	-
Clade K	*Leptogium*	OTU K11	JR10239C	Kenya, Taita Hills	Mwachora	MW335482	JX503885
Clade K	*Leptogium*	OTU K11	UK160548a	Tanzania, Mt. Kilimanjaro	Home 3	MW335483	MW340429
Clade K	*Leptogium*	OTU K11	UK170917b	Tanzania, Mt. Kilimanjaro	Flm6	MW335484	MW340430
Clade K	*Leptogium*	OTU K12	JR_Ar1	Kenya, Taita Hills	Ngangao	-	JX503889
Clade K	*Leptogium*	OTU K12	UK110511	Kenya, Taita Hills	Vuria	MW335485	JX503888
Clade K	*Leptogium*	OTU K12	UK110511b	Kenya, Taita Hills	Vuria	MW335486	-
Clade K	*Leptogium*	OTU K13	JR10218A	Kenya, Taita Hills	Yale	-	JX503886
Clade K	*Leptogium*	OTU K13	JR10218B	Kenya, Taita Hills	Yale	MW335487	JX503887
Clade K	*Leptogium*	OTU K13	UK170948d	Tanzania, Mt. Kilimanjaro	Flm1	MW335488	MW340431
Clade K	*Leptogium*	OTU K14	JR_D12_B2	Kenya, Taita Hills	Ngangao	-	JX503894
Clade K	*Leptogium*	OTU K14	JR_Z7B	Kenya, Taita Hills	Ngangao	-	JX503893
Clade K	*Leptogium*	OTU K14	JR10114A	Kenya, Taita Hills	Vuria	-	JX503891
Clade K	*Leptogium*	OTU K14	JR10134A	Kenya, Taita Hills	Ngangao	-	JX503890
Clade K	*Leptogium*	OTU K14	JR10136A	Kenya, Taita Hills	Ngangao	MW335489	JX503896
Clade K	*Leptogium*	OTU K14	JR10136B	Kenya, Taita Hills	Ngangao	-	JX503898
Clade K	*Leptogium*	OTU K14	JR10163Ab	Kenya, Taita Hills	Yale	MW335490	-
Clade K	*Leptogium*	OTU K14	JR10210B	Kenya, Taita Hills	Yale	MW335491	JX503892
Clade K	*Leptogium*	OTU K14	UK110505B	Kenya, Taita Hills	Ngangao	MW335492	JX503897
Clade K	*Leptogium*	OTU K14	UK110507	Kenya, Taita Hills	Ngangao	MW335493	JX503895
Clade L	*Leptogium*	OTU L1	UK170806g	Tanzania, Mt. Kilimanjaro	FPD4	MW335494	MW340432
Clade L	*Leptogium*	OTU L1	UK171320k	Tanzania, Mt. Kilimanjaro	FED1	MW335495	MW340433
Clade L	*Leptogium*	OTU L1	UK171468n	Tanzania, Mt. Kilimanjaro	FPD1	MW335496	MW340434
Clade L	*Leptogium*	OTU L1	UK171527e	Tanzania, Mt. Kilimanjaro	FPo2	MW335497	MW340435
Clade L	*Leptogium*	OTU L1	UK171585d	Tanzania, Mt. Kilimanjaro	FPo3	MW335498	MW340436
Clade L	*Leptogium*	OTU L2	JR10099	Kenya, Taita Hills	Vuria	MW335499	JX503781
Clade L	*Leptogium*	OTU L2	JR10161	Kenya, Taita Hills	Yale	MW335500	JX503782
Clade L	*Leptogium*	OTU L2	JR11087	Kenya, Taita Hills	Chawia	MW335501	JX503783
Clade L	*Leptogium*	OTU L2	UK170787e	Tanzania, Mt. Kilimanjaro	FOc2	MW335502	MW340437
Clade L	*Leptogium*	OTU L2	UK170801g	Tanzania, Mt. Kilimanjaro	FOc1	MW335503	MW340438
Clade L	*Leptogium*	OTU L2	UK170833f	Tanzania, Mt. Kilimanjaro	FPD4	MW335504	MW340439
Clade L	*Leptogium*	OTU L2	UK170845q	Tanzania, Mt. Kilimanjaro	FPD3	MW335505	MW340440
Clade L	*Leptogium*	OTU L2	UK170900e	Tanzania, Mt. Kilimanjaro	FOD4	MW335506	MW340441
Clade L	*Leptogium*	OTU L2	UK171494c	Tanzania, Mt. Kilimanjaro	FOc3	MW335507	-
Clade L	*Leptogium*	OTU L2	UK171507h	Tanzania, Mt. Kilimanjaro	FOD2	MW335508	MW340442
Clade L	*Leptogium*	OTU L2	UK171510n	Tanzania, Mt. Kilimanjaro	FOD2	MW335509	MW340443
Clade L	*Leptogium*	OTU L3	JR10152A	Kenya, Taita Hills	Yale	MW335510	JX503777
Clade L	*Leptogium*	OTU L3	JR10152B	Kenya, Taita Hills	Yale	MW335511	-
Clade L	*Leptogium*	OTU L3	JR10182A	Kenya, Taita Hills	Yale	MW335512	JX503776
Clade L	*Leptogium*	OTU L3	JR110094x	Kenya, Taita Hills	Chawia	MW335513	-
Clade L	*Leptogium*	OTU L3	JR11094B	Kenya, Taita Hills	Chawia	-	JX503775
Clade L	*Leptogium*	OTU L3	UK170804d	Tanzania, Mt. Kilimanjaro	FOc1	MW335514	MW340444
Clade L	*Leptogium*	OTU L3	UK170916am	Tanzania, Mt. Kilimanjaro	Flm6	MW335515	MW340445
Clade L	*Leptogium*	OTU L3	UK170933c	Tanzania, Mt. Kilimanjaro	Flm1	MW335516	MW340446

Clade			Collection number	Collection locality	Forest/plot	NCBI Accession No. mtSSU	nuITS
Clade L	*Leptogium*	OTU L3	UK170940a	Tanzania, Mt. Kilimanjaro	Flm1	MW335517	-
Clade L	*Leptogium*	OTU L3	UK171528c	Tanzania, Mt. Kilimanjaro	FPo2	MW335518	MW340447
Clade L	*Leptogium*	OTU L4	JR10K084Ab	Kenya, Mt. Kasigau	Kasigau N8-9	MW335519	MW340448
Clade L	*Leptogium*	OTU L4	JR10K084B	Kenya, Mt. Kasigau	Kasigau N8-9	MW335520	MW340449
Clade L	*Leptogium*	OTU L4	JR10K111a	Kenya, Mt. Kasigau	Kasigau N11	MW335521	MW340450
Clade L	*Leptogium*	OTU L4	JR290A	Kenya, Mt. Kasigau	Kasigau N-slope	MW335522	-
Clade L	*Leptogium*	OTU L4	JR290C	Kenya, Mt. Kasigau	Kasigau N-slope	MW335523	-
Clade L	*Leptogium*	OTU L5	UK160479f	Tanzania, Mt. Kilimanjaro	home 1	MW335524	-
Clade L	*Leptogium*	OTU L5	UK160552g	Tanzania, Mt. Kilimanjaro	home 3	MW335525	-
Clade L	*Leptogium*	OTU L5	UK170794k	Tanzania, Mt. Kilimanjaro	FOc2	MW335526	MW340451
Clade L	*Leptogium*	OTU L6	JR10149A	Kenya, Taita Hills	Yale	MW335527	JX503780
Clade L	*Leptogium*	OTU L6	JR10163C	Kenya, Taita Hills	Yale	MW335528	-
Clade L	*Leptogium*	OTU L6	JR10178A	Kenya, Taita Hills	Yale	MW335529	JX503779
Clade L	*Leptogium*	OTU L6	JR10279A	Kenya, Taita Hills	Macha	MW335530	JX503778
Clade L	*Leptogium*	OTU L6	JR10279B	Kenya, Taita Hills	Macha	MW335531	JX503778
Clade L	*Leptogium*	OTU L7	UK170852b	Tanzania, Mt. Kilimanjaro	FPo4	MW335532	MW340452
Clade L	*Leptogium*	OTU L7	UK170907x	Tanzania, Mt. Kilimanjaro	FOD5	MW335533	MW340453
Clade L	*Leptogium*	OTU L7	UK171185k	Tanzania, Mt. Kilimanjaro	FOD3	MW335534	MW340454
Clade L	*Leptogium*	OTU L7	UK171473h	Tanzania, Mt. Kilimanjaro	FPD1	MW335535	MW340455
Clade L	*Leptogium*	OTU L7	UK171478d	Tanzania, Mt. Kilimanjaro	FOc4	MW335536	MW340456
Clade L	*Leptogium*	OTU L7	UK171486k	Tanzania, Mt. Kilimanjaro	FOc4	MW335537	MW340457
Clade L	*Leptogium*	OTU L7	UK171514h	Tanzania, Mt. Kilimanjaro	FOc5	MW335538	MW340458
Clade L	*Leptogium*	OTU L7	UK171524c	Tanzania, Mt. Kilimanjaro	FPo2	MW335539	MW340459
Clade L	*Leptogium*	OTU L7	UK171587e	Tanzania, Mt. Kilimanjaro	FPo3	MW335540	MW340460
Clade L	*Leptogium*	OTU L7	UK171587f	Tanzania, Mt. Kilimanjaro	FPo3	MW335541	MW340461
Clade L	*Leptogium*	OTU L7	UK171591f	Tanzania, Mt. Kilimanjaro	FPo3	MW335542	MW340462
Clade M	*Leptogium*	OTU M1	JR10K101	Kenya, Mt. Kasigau	Kasigau N10	MW335543	MW340463
Clade M	*Leptogium*	OTU M1	JR10K241	Kenya, Mt. Kasigau	Kasigau E9	MW335544	MW340464
Clade M	*Leptogium*	OTU M1	JR10K451a	Kenya, Mt. Kasigau	Kasigau W1	MW335545	MW340465
Clade M	*Leptogium*	OTU M1	JR10K452a	Kenya, Mt. Kasigau	Kasigau W1	MW335546	MW340466
Clade M	*Leptogium*	OTU M1	JR10K479	Kenya, Mt. Kasigau	Kasigau W3-5	MW335547	MW340467
Clade M	*Leptogium*	OTU M1	JR10K493	Kenya, Mt. Kasigau	Kasigau W5	MW335548	MW340468
Clade N	*Leptogium*	OTU N1	UK170806e	Tanzania, Mt. Kilimanjaro	FPD4	MW335549	-
Clade N	*Leptogium*	OTU N1	UK170855c	Tanzania, Mt. Kilimanjaro	FPo4	MW335550	-
Clade N	*Leptogium*	OTU N1	UK170881h	Tanzania, Mt. Kilimanjaro	FPo5	MW335551	MW340469
Clade N	*Leptogium*	OTU N1	UK170882d	Tanzania, Mt. Kilimanjaro	FPo5	MW335552	MW340470
Clade N	*Leptogium*	OTU N1	UK170907v	Tanzania, Mt. Kilimanjaro	FOD5	MW335553	-
Clade N	*Leptogium*	OTU N1	UK170916b	Tanzania, Mt. Kilimanjaro	Flm6	MW335554	MW340471
Clade N	*Leptogium*	OTU N1	UK171486h	Tanzania, Mt. Kilimanjaro	FOc4	MW335555	MW340472
Clade N	*Leptogium*	OTU N1	UK171490c	Tanzania, Mt. Kilimanjaro	FOc3	MW335556	MW340473
Clade N	*Leptogium*	OTU N1	UK171504f	Tanzania, Mt. Kilimanjaro	FOD2	MW335557	MW340474
Clade N	*Leptogium*	OTU N1	UK171519n	Tanzania, Mt. Kilimanjaro	FOc5	MW335558	MW340475
Clade N	*Leptogium*	OTU N1	UK171527b	Tanzania, Mt. Kilimanjaro	FPo2	MW335559	-
Clade N	*Leptogium*	OTU N1	UK171582f	Tanzania, Mt. Kilimanjaro	FPD2	MW335560	MW340476
Clade N	*Leptogium*	OTU N1	UK171590k	Tanzania, Mt. Kilimanjaro	FPo3	MW335561	MW340477
Clade O	*Leptogium*	OTU O1	JR11004B	Kenya, Taita Hills	Werugha	MW335562	-
Clade O	*Leptogium*	OTU O1	UK160551b	Tanzania, Mt. Kilimanjaro	home 3	MW335563	MW340478
Clade P	*Leptogium*	OTU P1	JR10012	Kenya, Taita Hills	Taita Research Station	-	JX503899
Clade P	*Leptogium*	OTU P1	JR10021	Kenya, Taita Hills	Taita Research Station	-	JX503900
Clade P	*Leptogium*	OTU P1	JR10022A	Kenya, Taita Hills	Taita Research Station	MW335564	-
Clade P	*Leptogium*	OTU P1	JR10022B	Kenya, Taita Hills	Taita Research Station	-	JX503906
Clade P	*Leptogium*	OTU P1	JR10036	Kenya, Taita Hills	Shomoto Hill	-	JX503904
Clade P	*Leptogium*	OTU P1	JR10054	Kenya, Taita Hills	Shomoto Hill	-	JX503901
Clade P	*Leptogium*	OTU P1	JR10062	Kenya, Taita Hills	Shomoto Hill	MW335565	JX503902
Clade P	*Leptogium*	OTU P1	JR10063A	Kenya, Taita Hills	Shomoto Hill	-	JX503905
Clade P	*Leptogium*	OTU P1	JR10222B	Kenya, Taita Hills	Mwachora	-	JX503903
Clade P	*Leptogium*	OTU P1	JR10K091a	Kenya, Mt. Kasigau	Kasigau N9	MW335566	MW340479
Clade P	*Leptogium*	OTU P1	JR10K341b	Kenya, Mt. Kasigau	Kasigau S2	MW335567	MW340480
Clade P	*Leptogium*	OTU P1	JR10K392Aa	Kenya, Mt. Kasigau	Kasigau S7	MW335568	MW340481
Clade P	*Leptogium*	OTU P1	JR10K411	Kenya, Mt. Kasigau	Kasigau S41	MW335569	MW340482

Clade			Collection number	Collection locality	Forest/plot	NCBI Accession No. mtSSU	nuITS
Clade P	*Leptogium*	OTU P1	JR10K416a	Kenya, Mt. Kasigau	Kasigau S41	MW335570	MW340483
Clade P	*Leptogium*	OTU P1	JR10K421	Kenya, Mt. Kasigau	Kasigau S10	MW335571	MW340484
Clade P	*Leptogium*	OTU P1	UK160487a	Tanzania, Mt. Kilimanjaro	home 4	MW335572	MW340485
Clade P	*Leptogium*	OTU P1	UK160498a	Tanzania, Mt. Kilimanjaro	home 4	MW335573	-
Clade P	*Leptogium*	OTU P1	UK160557c	Tanzania, Mt. Kilimanjaro	home 3	MW335574	-
Clade P	*Leptogium*	OTU P1	UK160583a	Tanzania, Mt. Kilimanjaro	home 2	MW335575	MW340486
Clade P	*Leptogium*	OTU P1	UK170985a	Tanzania, Mt. Kilimanjaro	Home 5	MW335576	MW340487
Clade P	*Leptogium*	OTU P1	UK170998b	Tanzania, Mt. Kilimanjaro	Home 5	MW335577	MW340488
Clade P	*Leptogium*	OTU P1	UK171202a	Tanzania, Mt. Kilimanjaro	Flm3	MW335578	MW340489
Clade Q	*Leptogium*	OTU Q1	JR10019A	Kenya, Taita Hills	Taita Research Station	MW335579	-
Clade Q	*Leptogium*	OTU Q1	JR10045	Kenya, Taita Hills	Shomoto Hill	MW335580	-
Clade Q	*Leptogium*	OTU Q1	UK170880o	Tanzania, Mt. Kilimanjaro	FPo5	MW335581	MW340490
Clade Q	*Leptogium*	OTU Q1	UK170896c	Tanzania, Mt. Kilimanjaro	FPo5	MW335582	-
Clade Q	*Leptogium*	OTU Q1	UK170900d	Tanzania, Mt. Kilimanjaro	FOD4	MW335583	-
Clade Q	*Leptogium*	OTU Q1	UK170935q	Tanzania, Mt. Kilimanjaro	Flm1	MW335584	MW340491
Clade Q	*Leptogium*	OTU Q1	UK170936k	Tanzania, Mt. Kilimanjaro	Flm1	MW335585	MW340492
Clade Q	*Leptogium*	OTU Q1	UK170949b	Tanzania, Mt. Kilimanjaro	Flm1	MW335586	MW340493
Clade Q	*Leptogium*	OTU Q1	UK171185n	Tanzania, Mt. Kilimanjaro	FOD3	MW335587	MW340494
Clade Q	*Leptogium*	OTU Q1	UK171506f	Tanzania, Mt. Kilimanjaro	FOD2	MW335588	MW340495
Clade Q	*Leptogium*	OTU Q1	UK171510g	Tanzania, Mt. Kilimanjaro	FOD2	MW335589	MW340496
Clade Q	*Leptogium*	OTU Q1	UK171514j	Tanzania, Mt. Kilimanjaro	FOc5	MW335590	MW340497
Clade Q	*Leptogium*	OTU Q1	UK171521e	Tanzania, Mt. Kilimanjaro	FOc5	MW335591	-
Clade Q	*Leptogium*	OTU Q1	UK171590g	Tanzania, Mt. Kilimanjaro	FPo3	MW335592	MW340498
Clade Q	*Leptogium*	OTU Q2	JR10088A	Kenya, Taita Hills	Vuria	MW335593	-
Clade Q	*Leptogium*	OTU Q2	JR10114B	Kenya, Taita Hills	Vuria	MW335594	-
Clade Q	*Leptogium*	OTU Q2	UK170855e	Tanzania, Mt. Kilimanjaro	FPo4	MW335595	-
Clade Q	*Leptogium*	OTU Q2	UK171397b	Tanzania, Mt. Kilimanjaro	FEr4	MW335596	-
Clade Q	*Leptogium*	OTU Q2	UK171586v	Tanzania, Mt. Kilimanjaro	FPo3	MW335597	-
Clade Q	*Leptogium*	OTU Q2	UK171591b	Tanzania, Mt. Kilimanjaro	FPo3	MW335598	-
Clade Q	*Leptogium*	OTU Q3	UK170897a	Tanzania, Mt. Kilimanjaro	FOD4	MW335599	MW340499
Clade Q	*Leptogium*	OTU Q3	UK170940b	Tanzania, Mt. Kilimanjaro	Flm1	MW335600	MW340500
Clade Q	*Leptogium*	OTU Q4	UK170882a	Tanzania, Mt. Kilimanjaro	FPo5	MW335601	-
Clade Q	*Leptogium*	OTU Q4	UK170920d	Tanzania, Mt. Kilimanjaro	Flm6	MW335602	-
Clade Q	*Leptogium*	OTU Q4	UK171597a	Tanzania, Mt. Kilimanjaro	Flm2	MW335603	-
Clade R	*Leptogium*	sp.	JR_W1B_R2	Kenya, Mt. Kasigau	Kasigau	-	JX503907
Clade R	*Leptogium*	sp.	UK160559f	Tanzania, Mt. Kilimanjaro	Home 3	MW335604	-
Clade R	*Leptogium*	sp.	UK170881i	Tanzania, Mt. Kilimanjaro	FPo5	MW335605	MW340501
Clade R	*Leptogium*	sp.	UK170909b	Tanzania, Mt. Kilimanjaro	FOD5	MW335606	MW340502
Clade R	*Leptogium*	sp.	UK171204a	Tanzania, Mt. Kilimanjaro	Flm3	MW335607	MW340503
Clade R	*Leptogium*	OTU R1	JR10030B	Kenya, Taita Hills	Shomoto Hill	-	JX503925
Clade R	*Leptogium*	OTU R1	JR10030C	Kenya, Taita Hills	Shomoto Hill	MW335608	-
Clade R	*Leptogium*	OTU R1	JR10277B	Kenya, Taita Hills	Macha	MW335609	JX503927
Clade R	*Leptogium*	OTU R1	JR11001	Kenya, Taita Hills	Werugha	MW335610	JX503926
Clade R	*Leptogium*	OTU R1	JR11004A	Kenya, Taita Hills	Werugha	MW335611	JX503928
Clade R	*Leptogium*	OTU R2	JR10038B	Kenya, Taita Hills	Shomoto Hill	-	JX503924
Clade R	*Leptogium*	OTU R2	UK171189a	Tanzania, Mt. Kilimanjaro	Flm4	MW335612	MW340504
Clade R	*Leptogium*	OTU R3	JR10186B	Kenya, Taita Hills	Yale	-	JX503923
Clade R	*Leptogium*	OTU R3	UK170912g	Tanzania, Mt. Kilimanjaro	FOD5	MW335613	MW340505
Clade R	*Leptogium*	OTU R3	UK171482d	Tanzania, Mt. Kilimanjaro	FOc4	MW335614	MW340506
Clade R	*Leptogium*	OTU R3	UK171494e	Tanzania, Mt. Kilimanjaro	FOc3	MW335615	MW340507
Clade R	*Leptogium*	OTU R3	UK171494f	Tanzania, Mt. Kilimanjaro	FOc3	MW335616	MW340508
Clade R	*Leptogium*	OTU R3	UK171504g	Tanzania, Mt. Kilimanjaro	FOD2	MW335617	MW340509
Clade R	*Leptogium*	OTU R4	UK170845t	Tanzania, Mt. Kilimanjaro	FPD3	MW335618	MW340510
Clade R	*Leptogium*	OTU R4	UK170845u	Tanzania, Mt. Kilimanjaro	FPD3	MW335619	MW340511
Clade R	*Leptogium*	OTU R4	UK171320j	Tanzania, Mt. Kilimanjaro	FED1	MW335620	MW340512
Clade R	*Leptogium*	OTU R4	UK171469d	Tanzania, Mt. Kilimanjaro	FPD1	MW335621	MW340513
Clade R	*Leptogium*	OTU R4	UK171473f	Tanzania, Mt. Kilimanjaro	FPD1	MW335622	MW340514
Clade R	*Leptogium*	OTU R4	UK171527c	Tanzania, Mt. Kilimanjaro	FPo2	MW335623	MW340515
Clade R	*Leptogium*	OTU R4	UK171582n	Tanzania, Mt. Kilimanjaro	FPD2	MW335624	MW340516
Clade R	*Leptogium*	OTU R5	UK171320h	Tanzania, Mt. Kilimanjaro	FED1	MW335625	MW340517

Clade			Collection number	Collection locality	Forest/plot	NCBI Accession No. mtSSU	nuITS
Clade R	*Leptogium*	OTU R5	UK171590n	Tanzania, Mt. Kilimanjaro	FPo3	MW335626	MW340518
Clade R	*Leptogium*	OTU R6	JR10033B	Kenya, Taita Hills	Shomoto Hill	MW335627	JX503920
Clade R	*Leptogium*	OTU R6	JR10035	Kenya, Taita Hills	Shomoto Hill	MW335628	JX503919
Clade R	*Leptogium*	OTU R6	JR10040A	Kenya, Taita Hills	Shomoto Hill	-	JX503914
Clade R	*Leptogium*	OTU R6	JR10041Aa	Kenya, Taita Hills	Shomoto Hill	MW335629	JX503917
Clade R	*Leptogium*	OTU R6	JR10050	Kenya, Taita Hills	Shomoto Hill	-	JX503915
Clade R	*Leptogium*	OTU R6	JR10053C	Kenya, Taita Hills	Shomoto Hill	-	JX503916
Clade R	*Leptogium*	OTU R6	JR10206A	Kenya, Taita Hills	Yale	-	JX503909
Clade R	*Leptogium*	OTU R6	JR10206B	Kenya, Taita Hills	Yale	-	JX503910
Clade R	*Leptogium*	OTU R6	JR10260A	Kenya, Taita Hills	Mwachora	-	JX503911
Clade R	*Leptogium*	OTU R6	JR10277C	Kenya, Taita Hills	Macha	MW335630	-
Clade R	*Leptogium*	OTU R6	JR10279C	Kenya, Taita Hills	Macha	MW335631	JX503912
Clade R	*Leptogium*	OTU R6	JR10K531A	Kenya, Mt. Kasigau	Kasigau W9	MW335632	MW340519
Clade R	*Leptogium*	OTU R6	JR11009B	Kenya, Taita Hills	Vuria	-	JX503913
Clade R	*Leptogium*	OTU R6	JR11010B	Kenya, Taita Hills	Vuria	MW335633	JX503908
Clade R	*Leptogium*	OTU R6	JR11054E	Kenya, Taita Hills	Vuria	MW335634	-
Clade R	*Leptogium*	OTU R6	JR11088B	Kenya, Taita Hills	Chawia	-	JX503918
Clade R	*Leptogium*	OTU R6	UK170916a	Tanzania, Mt. Kilimanjaro	Flm6	MW335635	MW340520
Clade R	*Leptogium*	OTU R6	UK170927g	Tanzania, Mt. Kilimanjaro	Flm6	MW335636	MW340521
Clade R	*Leptogium*	OTU R6	UK171486g	Tanzania, Mt. Kilimanjaro	FOc4	MW335637	MW340522
Clade R	*Leptogium*	OTU R6	UK171486j	Tanzania, Mt. Kilimanjaro	FOc4	MW335638	MW340523
Clade R	*Leptogium*	OTU R7	JR_W5B	Kenya, Mt. Kasigau	Kasigau	MW335639	JX503922
Clade R	*Leptogium*	OTU R7	JR_W9	Kenya, Mt. Kasigau	Kasigau	MW335640	JX503921
Clade R	*Leptogium*	OTU R7	JR10K492a	Kenya, Mt. Kasigau	Kasigau W5	MW335641	MW340524
Clade R	*Leptogium*	OTU R8	UK160552d	Tanzania, Mt. Kilimanjaro	Home 3	MW335642	-
Clade R	*Leptogium*	OTU R8	UK170933e	Tanzania, Mt. Kilimanjaro	Flm1	MW335643	MW340525
Clade R	*Leptogium*	OTU R8	UK170950g	Tanzania, Mt. Kilimanjaro	Flm1	MW335644	MW340526
Clade R	*Leptogium*	OTU R9	UK171470b	Tanzania, Mt. Kilimanjaro	FPD1	MW335645	MW340527
Clade R	*Leptogium*	OTU R9	UK171519p	Tanzania, Mt. Kilimanjaro	FOc5	MW335646	MW340528
Clade R	*Leptogium*	OTU R9	UK171523e	Tanzania, Mt. Kilimanjaro	FPo2	MW335647	MW340529
Clade R	*Leptogium*	OTU R9	UK171584h	Tanzania, Mt. Kilimanjaro	FPD2	MW335648	MW340530
Clade R	*Leptogium*	OTU R9	UK171586ah	Tanzania, Mt. Kilimanjaro	FPo3	MW335649	MW340531
Clade R	*Leptogium*	OTU R9	UK171586u	Tanzania, Mt. Kilimanjaro	FPo3	MW335650	MW340532
Clade R	*Leptogium*	OTU R9	UK171587d	Tanzania, Mt. Kilimanjaro	FPo3	MW335651	MW340533
