# Supplementary figures and images for: Diversity of Leptogium (Collemataceae, Ascomycota) in East African Montane Ecosystems
Source: Microorganisms. 2021 Feb 3;9(2):314. doi: 10.3390/microorganisms9020314 (PMC7913733; doi:10.3390/microorganisms9020314)

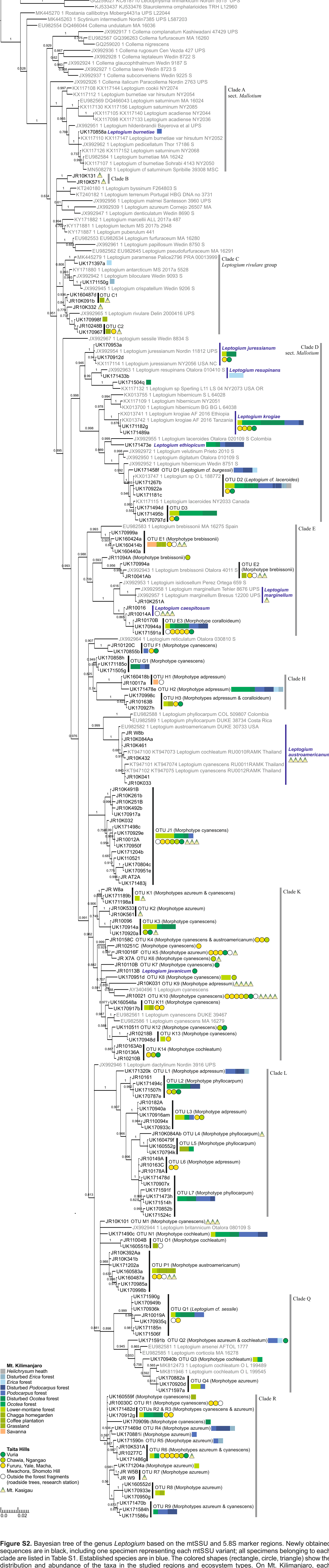

Supplement: Supplementary file 1 [file microorganisms-09-00314-s001.zip › Supplementary/FigureS2.pdf]
